# Supplementary material for: Parent values and preferences underpinning treatment decision making in poor prognosis childhood cancer: a scoping review protocol
Source: BMJ Open. 2021 May 13;11(5):e046284. doi: 10.1136/bmjopen-2020-046284 (PMC8126319; doi:10.1136/bmjopen-2020-046284)
Supplement: Author's manuscript [file bmjopen-2020-046284.draft_revisions.pdf]

BMJ Open is committed to open peer review. As part of this commitment we make the peer review history of every article we publish publicly available.

When an article is published we post the peer reviewers' comments and the authors' responses online. We also post the versions of the paper that were used during peer review. These are the versions that the peer review comments apply to.

The versions of the paper that follow are the versions that were submitted during the peer review process. They are not the versions of record or the final published versions. They should not be cited or distributed as the published version of this manuscript.

BMJ Open is an open access journal and the full, final, typeset and author-corrected version of record of the manuscript is available on our site with no access controls, subscription charges or pay-per-view fees (http://bmjopen.bmj.com).

If you have any questions on BMJ Open's open peer review process please email info.bmjopen@bmj.com

BMJ Open

Parent values and preferences underpinning treatment decision-making in poor-prognosis childhood cancer: A scoping review protocol

Journal:	*BMJ Open*
Manuscript ID	bmjopen-2020-046284
Article Type:	Protocol
Date Submitted by the Author:	26-Oct-2020
Complete List of Authors:	Pearson, Helen; Royal Marsden NHS Foundation Trust, ; University of Southampton Faculty of Medicine Health and Life Sciences,<br>Gibson, Faith; University of Surrey, Faculty of Health and Medical Sciences; Great Ormond Street Hospital For Children NHS Foundation Trust,<br>Darlington, Anne-Sophie; University of Southamptom, Faculty of Health Sciences
Keywords:	Paediatric oncology < PAEDIATRICS, Paediatric palliative care < PALLIATIVE CARE, Cancer pain < ONCOLOGY

SCHOLARONE™
Manuscripts

I, the Submitting Author has the right to grant and does grant on behalf of all authors of the Work (as defined in the below author licence), an exclusive licence and/or a non-exclusive licence for contributions from authors who are: i) UK Crown employees; ii) where BMJ has agreed a CC-BY licence shall apply, and/or iii) in accordance with the terms applicable for US Federal Government officers or employees acting as part of their official duties; on a worldwide, perpetual, irrevocable, royalty-free basis to BMJ Publishing Group Ltd ("BMJ") its licensees and where the relevant Journal is co-owned by BMJ to the co-owners of the Journal, to publish the Work in this journal and any other BMJ products and to exploit all rights, as set out in our licence.

The Submitting Author accepts and understands that any supply made under these terms is made by BMJ to the Submitting Author unless you are acting as an employee on behalf of your employer or a postgraduate student of an affiliated institution which is paying any applicable article publishing charge ("APC") for Open Access articles. Where the Submitting Author wishes to make the Work available on an Open Access basis (and intends to pay the relevant APC), the terms of reuse of such Open Access shall be governed by a Creative Commons licence – details of these licences and which Creative Commons licence will apply to this Work are set out in our licence referred to above.

Other than as permitted in any relevant BMJ Author's Self Archiving Policies, I confirm this Work has not been accepted for publication elsewhere, is not being considered for publication elsewhere and does not duplicate material already published. I confirm all authors consent to publication of this Work and authorise the granting of this licence.

For peer review only

Parent values and preferences underpinning treatment decision-making in poor-prognosis childhood cancer: A scoping review protocol

Helen Pearson[1,2] Professor Faith Gibson[3,4] Professor Anne-Sophie Darlington[1]

1. School of Health Sciences, University of Southampton, Southampton, United Kingdom
2. The Oak Centre for Children and Young People, The Royal Marsden NHS Foundation Trust, Sutton, Surrey, United Kingdom
3. Centre for Outcomes and Experience Research in Children's Health, Illness and Disability (ORCHID), Great Ormond Street Hospital for Children NHS Foundation Trust, London, United Kingdom
4. School of Health Sciences, University of Surrey, Guildford, Surrey, United Kingdom

Corresponding Author:

Helen Pearson

Helenpearson1@nhs.net

Word count: 2830

Keywords:

Cancer; Child; Decision-making; Parent; Poor-Prognosis

For peer review only - http://bmjopen.bmj.com/site/about/guidelines.xhtml

ABSTRACT

Objective:

The objective of this scoping review is to explore parent values and preferences underpinning treatment decision-making when their child is receiving cancer-directed therapy for a poor-prognosis cancer.

Introduction:

Parents of a child with cancer want to be involved in making treatment decisions for their child. Underpinning and informing these decisions are parents' individual values and preferences. Parents of a child who has a poor-prognosis cancer and who subsequently dies can experience decisional regret. To support parents, and potentially reduce decisional regret, identifying the values and preferences of parents who are making these treatment decisions may enhance the support which can be provided by healthcare professionals. An increased understanding will support future work in this area and identify research gaps which could strengthen support strategies in clinical practice.

Inclusion Criteria:

Research studies, literature and systematic reviews will be included. Articles written in English, from any country and published from 1995 to present will be considered.

Methods and Analysis:

The JBI scoping review methodology will be followed. An initial database search of CINHAL and Medline will be conducted to analyze the keywords using subject headings and MeSH terms. Articles will be initially screened on title and abstract. The reference and citation lists of the full-text articles to be included will be searched using Web of Science. Articles will be independently reviewed by two reviewers and any discrepancies discussed with a third reviewer. Data extracted will be presented in tabular, diagrams and descriptive summaries.

Ethics and Dissemination:

Ethical approval is not required for this scoping review. This review will inform further research with parents to understand their values and preferences when making repeated treatment decisions when their child has a poor-prognosis cancer. All outputs will be disseminated through peer-reviewed publications and conference presentations.

STRENGHS AND LIMTITATIONS OF THIS STUDY

- This is the first scoping review to identify parent values and preferences in treatment decision-making when their child has a poor-prognosis cancer
- Clear definitions for values and preferences and poor-prognosis are provided to support the reader in understanding the context of this scoping review
- The search strategy includes the reference and citation lists of all included full-text articles for a comprehensive search and cross-checking purposes

For peer review only - http://bmjopen.bmj.com/site/about/guidelines.xhtml

- There is no formal quality assessment of research literature reviewed in a scoping review. A narrative synthesis of the research literature reviewed within this scoping review will be undertaken and published.

For peer review only

For peer review only - http://bmjopen.bmj.com/site/about/guidelines.xhtml

INTRODUCTION

Globally approximately 300,000 children and young people from birth to nineteen years of age are diagnosed with cancer each year[1] and 90,000 of these children die from the disease.[2] Within the United Kingdom (UK) of the 1,420 children under the age of fifteen diagnosed with cancer every year,[3] roughly 250 children will die.[4] Parent involvement in their child's care can include making decisions regarding treatments, administering medications and advocating for their child within the multidisciplinary team. Research has shown that parents wants to be involved in all aspects of their child's care particularly when it comes to making treatment decisions.[5] However, parents are rarely involved in decision-making with initial treatment as this is protocol driven, with reduced choices, but take a more active role if treatments fail and relapse strategies are required.[6]

Studies investigating parental experiences of having a child with cancer have tended to focus on diagnoses such as leukemia or lymphoma, which generally have a good prognosis.[3] Parents of children with a poor-prognosis cancer are under-represented. The needs and experiences of this parent population are likely to be different because: 1) survival rates are lower;[3] 2) the number of children diagnosed with a cancer which has a poor-prognosis is lower;[3] 3) established treatment protocols are not always available due to low numbers of children being diagnosed, resulting in a longer time for clinical trials to become established.

Clinical trials within pediatric oncology have advanced over the past decade resulting in more treatment options for children with a poor-prognosis cancer. There has been a vast number of clinical trials involving immunotherapy and targeted inhibitors which have shown to improve or extend survival and in some cases provide cure within adult cancer.[7-8] As a result, these have been extended into clinical trials for children particularly for those who have a poor-prognosis cancer.[9-10] Where established treatment protocols may not be available, parents subsequently become involved in treatment decision-making including options such as clinical trials and off-protocol treatments. Often parents can make a series of repeated treatment decisions depending on their child's response to treatment and toxicities experienced.

Prognosis relates to the outcome of the cancer diagnosis and whether the disease is likely to return.[11] A poor-prognosis indicates a highly unfavorable treatment outcome likely to result in death. Emerging research has shown that 73% of parents whose child has a poor-prognosis and subsequently dies experience regret.[12] Regret is predominately related to the decisions parents have made and this regret can result in prolonged grieving.[13]

Research has acknowledged that parents pursue cancer-directed treatments even when the chance of cure is small.[14] Quality information including treatment options, risks and benefits, expectations of treatment and cure, to make informed decisions based on parent individual values and preferences is paramount.[5] Research on parent treatment decision-making at the end-of-life or opting to participate in phase I clinical trials has shown parent preferences for cancer-directed treatment includes hoping

For peer review only - http://bmjopen.bmj.com/site/about/guidelines.xhtml
For peer review only

for a cure, prolong life, decreasing suffering[5,14] and ensure they have done everything possible to save their child's life.[14] With the recognition that parents experience high levels of regret, and want to be involved in treatment decision-making, particularly when their child has a poor-prognosis, this requires an understanding of individual parent values and preferences which inform these decisions.

Values and preferences relate to the expectations, goals, beliefs and predispositions of an individual when making a decision.[15]These values and preferences inform the decision-making process for a person and ultimately define what decision they make. Supporting parents in understanding their own values and preferences and how these inform treatment decision-making is essential to help reduce regret. Support tools to elicit parent values and preferences can help in the process of making decisions.[16]As regret in this parent population is centered on treatment decision-making, understanding parent values and preferences may provide insight into what parents require in terms of support to enable them to make informed decisions.

This scoping review has been prompted by the author's clinical experience of caring for children receiving cancer-directed therapy for a poor-prognosis cancer. Parents are involved in making treatment decisions for their child, often with the need to make a series of repeated treatment decisions as their child's condition changes. This raises questions such as: What are parent values and preferences when their child is receiving cancer-directed therapy for a poor-prognosis cancer? How do these values and preferences inform treatment decision-making?

A preliminary search on MEDLINE using the EBSCO platform in April 2020 found two relevant reviews. A literature review published in 2013 investigated parent decision-making of care and treatment in childhood cancer.[17] This review did not specifically focus on parent values and preferences when their child has a poor-prognosis cancer where decision-making may differ due to the child's condition and likely outcome. It cannot be assumed that values and preferences would be the same for parents regardless of the outcome for their child (survival, death, disabilities as a result of diagnosis/treatment). This warrants specific investigation. A mixed-methods systematic review published in 2014 looked at medico-legal practice in judgements and decisions when a child's treatment is not curative.[18] The outcome from this review provides a comprehensive theoretical framework on the processes of parent decision-making. The framework focuses on transition from cancer-directed therapy to palliative/End-of-Life care for their child. How these decisions relate to individual values and preferences which underpins parents' decision-making, is not included in this framework. The framework also considers the illness trajectory from cancer-directed therapy to palliative/End-of-Life Care to be a linear process, which in clinical practice is not always the case.

This scoping review allows for a primary focus on parent values and preferences when their child is receiving cancer-directed therapy for a poor-prognosis cancer; this has not been specifically addressed previously. This review seeks to map the available research literature of parent values and preferences underpinning treatment decision-making when their child is receiving cancer-directed

For peer review only - http://bmjopen.bmj.com/site/about/guidelines.xhtml

therapy for a poor-prognosis cancer. Through clarifying these values and preferences, this scoping review will inform further research with parents who are making repeated treatment decisions when their child has a poor-prognosis cancer. Combining the data from this scoping review and data from parents who are making repeated treatment decisions, this will inform the development of a decision aid which is underpinned by parent values and preferences to support parents when making treatment decisions. Furthermore, the scoping review may identify research gaps which could be addressed in the future to strengthen the support given to parent when making treatment decisions for their child.

REVIEW OBJECTIVES AND QUESTIONS

The objective is to explore parent values and preferences underpinning treatment decision-making when their child is receiving cancer-directed therapy for a poor-prognosis cancer. This scoping review will seek to answer two questions:

1) What are parent values and preferences when their child is receiving cancer-directed therapy for a poor-prognosis cancer?
2) How do these values and preferences inform treatment decision-making?

INCLUSION CRITERIA

Participants

This will be parents of a child (<18 years) who has a poor-prognosis cancer and is receiving cancer-directed therapy.

Concept

The aim, is to describe, explore and understand the values and preferences which underpin parent treatment decision-making when their child is receiving cancer-directed therapy for a poor-prognosis cancer. The review will specifically focus on studies where the child is receiving cancer-directed therapy, defined as any type of cancer treatment as opposed to end-of-life care, palliative care or symptom management alone. The parent of a child who is receiving cancer-directed therapy and palliative care/symptom management will be included.

Context

Parents of a child who is receiving cancer-directed therapy for a poor-prognosis cancer in any clinical or medical setting (hospital, community and hospice) will be considered.

Types of evidence sources

International evidence sources for this review will be considered. As this is a small parent population within childhood cancer, international collaboration to enhance knowledge and progress research within this field is well established. Research studies, literature or systematic reviews of qualitative and quantitative nature will be included. Literature or systematic reviews which report the same study population as research studies included will be considered eligible as long as different data is presented. Otherwise these articles will be included and used for cross-checking purposes.

For peer review only - http://bmjopen.bmj.com/site/about/guidelines.xhtml

Studies included can be prospective or retrospective. Including retrospective studies is important due to the sensitivity of the topic as research has been carried out with parents after their child has died to reduce burden prospectively.

The search will include the last 25 years from 1995 to 2020 inclusive (until the time the database searches take place). Being a small parent population, research can take years to complete due to low participant numbers therefore an extensive search period is required.

METHODS

The Joanna Briggs Institute scoping review methodology will be followed.[19] This scoping review was registered on the Open Science Framework on 2nd June 2020 (https://osf.io/n7j9f). For the scoping review report, the Preferred Reporting Items for Systematic Reviews and Meta-Analyses (PRISMA-ScR) Checklist[20] will be followed.

SEARCH STRATEGY

Database searches will ascertain published research studies, literature or systematic reviews. An initial search was completed in Medline and Cumulative Index of Nursing and Allied Health Literature (CINAHL) using the EBSCO platform in April 2020. This initial database search found relevant primary studies using qualitative and quantitative methods and therefore warrants the scoping review being conducted. The key words were devised from known terminology used in clinical practice, academia, definitions (written in the introduction) and literature read on parent decision-making in childhood cancer. Table 1 shows the list of key words and phrases. Each key word was searched for subject headings/MeSH terms to allow for full exploration of the academic language associated with the word. Phrases were not searched for subject headings/MeSH terms as these are natural language phrases as opposed to academic language. The Medline search strategy is detailed in figure 1. From the articles retrieved in this initial search, the text words in the title and abstract were analyzed in combination with the index terms used to describe the articles. This did not provide any additional key words or phrases to be included in the database searches.

Table 1: Keywords for Subject Headings/MeSH Terms and Phrases for Search

Keywords searched for Subject Headings/MeSH Terms
Parent
Family
Value
Preference
Choice
Choose
Belief
Attitude
Expectation

Keyword	Relevant Phrases
Values & Preferences	"goal* of care"
Poor Prognosis	"advanc* cancer"
	"no realistic chance of cure"
	"disease* progress*"
	"palliat* chemotherap*"
	"no reasonable chance of cure"
Decision Making	"decision making"
Cancer	"Cancer-direct* therap"

For peer review only - http://bmjopen.bmj.com/site/about/guidelines.xhtml

Predisposition
Influence
Experience
Perception
Poor Prognosis
Relapse
Recurrent
Refractory
Incurable
Advanced Cancer
Uncertainty
Deteriorate
Disease progression
Cancer
Oncology
neoplasm
Malignancy
Child
Paediatric
Pediatric
Decision making

The databases for this review will be CINAHL, Medline and PsycINFO using the EBSCO platform and the Web of science core collections using the Clarivate platform. The list of key words will be searched for subject headings/MeSH terms separately in each of the databases with the acknowledgement that databases do not have a consistent approach to indexing articles. Phrases will not be searched for subject headings/MeSH terms. The use of search symbols such as truncation, adjacency and the wildcard will allow for full exploration of words. For example, p#ediatric provides inclusion of English and United States spelling of the word. Exact phrase searching with the use of quotation marks for example "decision-making" and "no realistic chance of cure" will be incorporated. This allows for inclusion of some commonly used phrases in clinical practice and within the research literature previously read. Boolean operators (AND/OR) will support the combination between key words, subject headings/MeSH terms using OR and the main topics using AND (parent, values and preferences, decision making, child, cancer, poor-prognosis).

Inclusion criteria for the review includes: published research studies, literature or systematic reviews written in English with full abstract available from January 1995 onwards. Articles can be from any country. Only articles written in English will be included due to the feasibility of being able to transcribe research published in other languages and the time/financial cost in doing so. Articles will

For peer review only - http://bmjopen.bmj.com/site/about/guidelines.xhtml

be initially screened on the title and then refined through reading the abstract before retrieving the full-text to confirm inclusion or exclusion. The Web of Science will be used to look at the reference and citation lists of all included full-text articles. This will highlight any additional articles not included in the database searches which may need to be considered for inclusion. Including citation lists is known as snowballing (or pearl growing) and considered an important aspect to ensure inclusivity of all relevant articles.[21] Gray literature will not be included as this is a niche topic with established international collaborations resulting in available research and literature reviews being published. The corresponding author for any article will be contacted if clarification is required for completeness of either including or excluding the article within the scoping review. The database searches will be peer-reviewed by the University Research Librarian for transparency and robustness of the search strategy.

SOURCE OF EVIDENCE SELECTION

Articles obtained will be stored on EndNote X9.2 and duplications removed. Initial screening of the studies will be done by reading the title and abstract to ascertain whether they fit the inclusion criteria. This screening will be carried out independently by two reviewers before the review team meet to discuss the articles to be included for full-text review. Any discrepancies will be resolved with the addition of the third reviewer.

Full-text of the articles included based on abstract will be retrieved. These will be read in full by two reviewers. Corresponding authors of the retrieved articles will be contacted for clarification if required. The review team will meet again to discuss the final articles to be included in the scoping review and the third reviewer will resolve any discrepancies. Any full-text articles which are excluded at this stage will be justified in the scoping review report. For transparency the search results will be presented in the PRISMA-ScR flowchart[20] which will systematically detail the selection process of articles.

DATA EXTRACTION

Data will be extracted from the articles obtained which meet the objectives of the scoping review. Data extraction will be undertaken in full by two reviewers and the third reviewer will randomly select 25% of the eligible articles to review. Once the data extraction has been completed independently by all three reviewers, the review team will meet to discuss and address any discrepancies.

For a consistent approach between reviewers, the Joanna Briggs Institute source of evidence template[19] will be modified to extract information, characteristics and findings from the articles which are relevant to answering the scoping review questions. This will include information on the population, concept, context, type of evidence source, study methods, values and preferences, information on what cancer-directed therapy was given and whether this was in conjunction with palliative care/symptom management. The modified data extraction template for this scoping review can be found in figure 2. Any amendments to the extraction template during the scoping review will be highlighted in the scoping review report.

For peer review only

For peer review only - http://bmjopen.bmj.com/site/about/guidelines.xhtml

ANALYSIS OF THE EVIDENCE

Analysis will commence with the final number of articles included in the scoping review and the basic characteristics of these articles. These characteristics will include the articles' first author, year of publication, country, study design and research methods, and the number of participants in each study.

The data extraction template (figure 2) will highlight the most consistent and clear way for the evidence to be presented which is representative of the extracted data once the scoping review is complete. For qualitative data, content analysis will provide a description of data extracted and will be mapped to show consistencies and differences amongst the data.

For quantitative data, the frequency and numerical information will be presented based on the data extracted. In both the qualitative and quantitative data extraction, the analysis will reflect the objectives for the scoping review.

PRESENTATION OF RESULTS

Results will be presented in formats such as tabular, diagrammatic and descriptive summaries to clearly represent the extracted data relevant to the scoping review objectives. The basic characteristics of the studies will be presented in table format. This will provide the reader with an overall summary of the studies which are included in the scoping review. Descriptive summaries of the qualitative data will be supported with visual diagrams mapping the relevant categories of the scoping review objectives. Quantitative data will be presented in graphs and figures to show comparisons and differences between the data extracted. The presentation of results may be refined during the scoping review to align with the data extracted from the included articles. Amendments to the suggested presentation will be highlighted in the scoping review report.

The scoping review will identify any research gaps which could enhance how support is provided to this parent population. Recommendations for practice which may support parents when making treatment decisions for cancer-directed therapy when their child has a poor-prognosis cancer will be highlighted.

ETHICS AND DISSEMINATION:

This scoping review is the first to look at parent values and preferences in treatment decision-making when their child has a poor-prognosis cancer. The results from this scoping review will be used to inform an interview schedule with parents to understand their values and preferences when making repeated treatment decisions for their child with relapse neuroblastoma, a poor prognosis childhood cancer. The combined results from this scoping review and parent interviews will inform the development of a decision aid to support parents in their treatment decision-making. This data and the decision aid developed will be published in peer-reviewed journals and presented at

For peer review only

national/international conferences consisting of healthcare professionals, charities and parents. This scoping review does not require ethical approval.

For peer review only

For peer review only - http://bmjopen.bmj.com/site/about/guidelines.xhtml

REFERENCES

1. Steliarova-Foucher E, Colombet M, Ries LAG, et al. International incidence of childhood cancer, 2001-10: a population-based registry study. *Lancet Oncol* 2017;18(6):719-731. doi: 10.1016/S1470-2045(17)30186-9 [published Online first: 11 April 2017].
2. Childhood Cancer International. [Internet] 2020 [cited 2020 May 30]. Available from: https://www.childhoodcancerinternational.org/
3. Public Health England. Childhood Cancer Statistics, England Annual Report 2018. [Internet] 2018 [cited 2020 Apr 28]. Available from: http://www.ncin.org.uk/publications/reports
4. Cancer Research UK. Children's cancer statistics. [Internet] 2019 [cited 2020 Apr 28]. Available from:https://www.cancerresearchuk.org/health-professional/cancer-statistics/childrens-cancers
5. Mack JW, Feudtner C, Hinds PS. Communication and decision support for children with advanced cancer and their families. *Am Soc Clin Oncol Educ Book*. 2012;32:637-43. doi: 10.14694/EdBook_AM.2012.32.164. [published Online first: 17 May, 2012].
6. Hinds PS, Oakes L, Furman W, et al. Decision making by parents and healthcare professionals when considering continued care for pediatric patients with cancer. *Oncol Nurs Forum* 1997;24(9):1523-28.
7. Lynch A, Murphy A. The emerging role of immunotherapy in colorectal cancer. *Ann Transl Med* 2016;4(16). doi: 10.21037/atm.2016.08.29.
8. Somasundaram A, Burns TF. The next generation of immunotherapy: keeping lung cancer in check. *J Hematol Oncol* 2017;10(1):87. doi: 10.1186/s13045-017-0456-5.
9. Mackall CL, Merchant MS, Fry TJ. Immune-based therapies for childhood cancer. *Nat Rev Clin Oncol* 2014;11:693–703. doi: 10.1186/s13045-017-0456-5. [published Online first: 28 October 2014].
10. Roberts SS, Chou AJ, Cheung N-KV. Immunotherapy of childhood sarcomas. *Front Oncol* 2015;5:181. doi: 10.3389/fonc.2015.00181.
11. National Cancer Institute. Understanding Cancer Prognosis. [Internet] 2019 [cited 2020 Mar 31]. Avaliable from: https://www.cancer.gov/about-cancer/diagnosis-staging/prognosis
12. Lichtenthal WG, Roberts KE, Catarozoli C, et al. Regret and unfinished business in parents bereaved by cancer: A mixed methods study. *J Palliat Med* 2020;34(3):367-377. doi: 10.1177/0269216319900301. [published Online first: 5 February 2020].
13. Lannen PK, Wolfe K, Prigerson HG, et al. Unresolved grief in a national sample of bereaved parents: impaired mental and physical health 4 to 9 years later. *J Clin Oncol* 2008;26(36):5870-5876. doi: 10.1200/JCO.2007.14.6738. [published Online first: 24 November 2008].
14. Tomlinson D, Bartels U, Gammon J, et al. Chemotherapy versus supportive care alone in pediatric palliative care for cancer: comparing the preferences of parents and healthcare professionals. *Can Med Assoc J* 2011;183(7):E1252-58. doi: 10.1503/cmaj.110392. [published Online first: 17 October 2011].

For peer review only

For peer review only - http://bmjopen.bmj.com/site/about/guidelines.xhtml

15. Straus SE, Richardson WS, Glasziou P, et al. Evidence-Based Medicine How to Practice and Teach EBM. 3rd ed. Edinburgh: Elsevier Churchill Livingstone 2005:12.
16. Marron JM, Mack JW. When to say when: How aggressively to care for children with multiply relapsed cancer?. *Pediatr* Blood Cancer 2015;62(7):1119-20. doi: 10.1002/pbc.25476. [published Online first: 8 March 2015].
17. Markward MJ, Benner K, Freese R. Perspectives of parents on making decisions about the care and treatment of a child with cancer: A review of the literature. *Fam Syst Health* 2013;31(4):406-413. doi: 10.1037/a0034440.
18. Valdez-Martinez E, Noyes J, Bedolla M. When to stop? Decision-making when children's cancer treatment is no longer curative: a mixed methods systematic review. *BMC Pediatr* 2014;4(24):124. doi: 10.1186/1471-2431-14-124.
19. Peters MDJ, Godfrey C, McInerney P, et al. Chapter 11: Scoping Reviews (2020 version). In: Aromataris E, Munn Z. (Editors) Joanna Briggs Institute Reviewer's Manual, JBI, 2020. Available from: https://reviewersmanual.joannabriggs.org/
20. Tricco, AC, Lillie E, Zarin W, et al. PRISMA Extension for Scoping Reviews (PRISMA-ScR): Checklist and Explanation. *Ann Intern Med* 2018;169(7): 467-73. doi: 10.7326/M18-0850. [published Online first: 4 September 2018].
21. Greenhalgn T, Peacock R. Effectiveness and efficiency of search methods in systematic reviews of complex evidence: audit of primary sources. *BMJ* 2005; 331:1064-1065. doi: 10.1136/bmj.38636.593461.68. [published Online first: 17 October 2005].

PATIENT AND PUBLIC INVOLVEMENT

No patient involvement for this scoping review.

AUTHOR CONTRIBUTIONS

HP, FG, ASD conceived the idea for this scoping review. HP developed the research questions, objectives, inclusion criteria, undertook initial database search, drafted and edited this manuscript. FG and ASD reviewed inclusion criteria, screened abstracts and full-text papers from initial database search, and edited this manuscript. HP, FG, ASD approved final manuscript version.

FUNDING STATEMENT:

This research received no specific grant from any funding agency in the public, commercial or not-for-profit sectors'

COMPETING INTEREST STATEMENT

None declared

FIGURE LEGENDS

Figure 1: Medline Search Strategy

Figure 2: JBI Data Extraction Tool

For peer review only - http://bmjopen.bmj.com/site/about/guidelines.xhtml

For peer review only

For peer review only - http://bmjopen.bmj.com/site/about/guidelines.xhtml

Figure 1: Medline Search Strategy

Medline Search Strategy	
S1	parent*
S2	mother*
S3	father*
S4	famil*
S5	S1 OR S2 OR S3 OR S4
S6	value*
S7	(MH "Value of Life") OR (MH "Social Values")
S8	preference*
S9	"goal* of care"
S10	choice*
S11	belief*
S12	(MH "Social Norms")
S13	attitude*
S14	expect*
S15	(MH "Motivation")
S16	predisposition*
S17	influenc*
S18	experienc*
S19	(MH "Clinical Decision-Making")
S20	choose*
S21	percept*
S22	S6 OR S7 OR S8 OR S9 OR S10 OR S11 OR S12 OR S13 OR S14 OR S15 OR S16 OR S17 OR S18 OR S19 OR S20 OR S21

For peer review only

S23 "decision making"
S24 (MH "Decision Making, Shared")
S25 decision N2 making
S26 decision*
S27 decide*
S28 S23 OR S24 OR S25 OR S26 OR S27
S29 S5 AND S22 AND S28
S30 "poor prognosis"
S31 relaps*
S32 (MH "Recurrence")
S33 recurrenc*
S34 refractory
S35 incurable
S36 (MH "Terminally Ill")
S37 "advanc* cancer"
S38 "no realistic chance of cure"
S39 uncertain*
S40 deteriorat*
S41 (MH "Clinical Deterioration")
S42 "disease* progress*"
S43 "palliat* chemotherap*"
S44 "no reasonable chance of cure"
S45 S30 OR S31 OR S32 OR S33 OR S34 OR S35 OR S36 OR S37 OR S38 OR S39 OR S40 OR S41 OR S42 OR S43 OR S44
S46 child*

For peer review only

S47	p#ediatric*
S48	S46 OR S47
S49	cancer
S50	neoplasm*
S51	(MH "Neoplasm Recurrence, Local")
S52	malignan*
S53	(MH "Neoplasms")
S54	"cancer direct* therap*"
S55	oncolog*
S56	S49 OR S50 OR S51 OR S52 OR S53 OR S54 OR S55
S57	S45 AND S48 AND S56
S58	S29 AND S57

For peer review only

Figure 2: JBI Data Extraction Template amended for this Scoping Review

Scoping Review Details	
Scoping Review Title	
Review Objectives	
Review Questions	
Inclusion/Exclusion Criteria	
Population (parent of a child under age of 18 years)	
Concept (parent of a child receiving cancer-directed therapy for a poor prognosis cancer)	
Context (hospital, community, hospice)	
Types of Evidence Source (Qual/Quant, mixed methods)	
Evidence source Details and Characteristics	
Citation details (author, year of publication, journal)	
Country	
Study Aims/Objectives	
Prospective or Retrospective study	

For peer review only

Research Methodology & Methods	
Participant Details (mother,father sample size)	
Data extracted from sources of evidence	
Cancer-directed therapy options available (list)	
Cancer-directed therapy in conjunction with palliative care/symptoms management	YES / NO
Cancer-directed therapy given to child (list if more than one)	
Outcome from cancer-directed therapy given	
Values and preferences identified (list)	
Explanation of how these values and preferences informed decision-making (explicitly stated/not stated/unsure)	
Article recommendations for future research/clinical practice	

For peer review only

BMJ Open

Parent values and preferences underpinning treatment decision-making in poor-prognosis childhood cancer: A scoping review protocol

Journal:	*BMJ Open*
Manuscript ID	bmjopen-2020-046284.R1
Article Type:	Protocol
Date Submitted by the Author:	16-Mar-2021
Complete List of Authors:	Pearson, Helen; Royal Marsden NHS Foundation Trust, ; University of Southampton Faculty of Medicine Health and Life Sciences,<br>Gibson, Faith; University of Surrey, Faculty of Health and Medical Sciences; Great Ormond Street Hospital For Children NHS Foundation Trust,<br>Darlington, Anne-Sophie; University of Southamptom, Faculty of Health Sciences
**Primary Subject Heading**:	Paediatrics
Secondary Subject Heading:	Oncology
Keywords:	Paediatric oncology < PAEDIATRICS, Paediatric palliative care < PALLIATIVE CARE, Cancer pain < ONCOLOGY

SCHOLARONE™
Manuscripts

BMJ

I, the Submitting Author has the right to grant and does grant on behalf of all authors of the Work (as defined in the below author licence), an exclusive licence and/or a non-exclusive licence for contributions from authors who are: i) UK Crown employees; ii) where BMJ has agreed a CC-BY licence shall apply, and/or iii) in accordance with the terms applicable for US Federal Government officers or employees acting as part of their official duties; on a worldwide, perpetual, irrevocable, royalty-free basis to BMJ Publishing Group Ltd ("BMJ") its licensees and where the relevant Journal is co-owned by BMJ to the co-owners of the Journal, to publish the Work in this journal and any other BMJ products and to exploit all rights, as set out in our licence.

The Submitting Author accepts and understands that any supply made under these terms is made by BMJ to the Submitting Author unless you are acting as an employee on behalf of your employer or a postgraduate student of an affiliated institution which is paying any applicable article publishing charge ("APC") for Open Access articles. Where the Submitting Author wishes to make the Work available on an Open Access basis (and intends to pay the relevant APC), the terms of reuse of such Open Access shall be governed by a Creative Commons licence – details of these licences and which Creative Commons licence will apply to this Work are set out in our licence referred to above.

Other than as permitted in any relevant BMJ Author's Self Archiving Policies, I confirm this Work has not been accepted for publication elsewhere, is not being considered for publication elsewhere and does not duplicate material already published. I confirm all authors consent to publication of this Work and authorise the granting of this licence.

For peer review only

Parent values and preferences underpinning treatment decision-making in poor-prognosis childhood cancer: A scoping review protocol

Helen Pearson[1,2] Professor Faith Gibson[3,4] Professor Anne-Sophie Darlington[1]

1. School of Health Sciences, University of Southampton, Southampton, United Kingdom
2. The Oak Centre for Children and Young People, The Royal Marsden NHS Foundation Trust, Sutton, Surrey, United Kingdom
3. Centre for Outcomes and Experience Research in Children's Health, Illness and Disability (ORCHID), Great Ormond Street Hospital for Children NHS Foundation Trust, London, United Kingdom
4. School of Health Sciences, University of Surrey, Guildford, Surrey, United Kingdom

Corresponding Author:
Helen Pearson
Helenpearson1@nhs.net

Word count: 2830

Keywords:
Cancer; Child; Decision-making; Parent; Poor-Prognosis

For peer review only

ABSTRACT

Introduction:

Parents of a child with cancer want to be involved in making treatment decisions for their child. Underpinning and informing these decisions are parents' individual values and preferences. Parents of a child who has a poor-prognosis cancer and who subsequently dies can experience decisional regret. To support parents, and potentially reduce decisional regret, identifying the values and preferences of parents who are making these treatment decisions may enhance the support which can be provided by healthcare professionals. An increased understanding will support future work in this area and identify research gaps which could strengthen support strategies in clinical practice. This scoping review is to explore parent values and preferences underpinning treatment decision-making when their child is receiving cancer-directed therapy for a poor-prognosis cancer.

Methods and Analysis:

The JBI scoping review methodology will be followed. An initial database search of CINHAL and Medline will be conducted to analyze the keywords using subject headings and MeSH terms. Articles will be initially screened on title and abstract. The reference and citation lists of the full-text articles to be included will be searched using Web of Science. Articles will be independently reviewed by two reviewers and any discrepancies discussed with a third reviewer. Data extracted will be presented in tabular, diagrams and descriptive summaries.

Ethics and Dissemination:

Ethical approval is not required for this scoping review. This review will inform further research with parents to understand their values and preferences when making repeated treatment decisions when their child has a poor-prognosis cancer. All outputs will be disseminated through peer-reviewed publications and conference presentations.

This scoping review is registered on the Open Science Framework (https://osf.io/n7j9f).

STRENGHS AND LIMTITATIONS OF THIS STUDY

- This is the first scoping review to identify parent values and preferences in treatment decision-making when their child has a poor-prognosis cancer
- The search strategy includes the reference and citation lists of all included full-text articles for a comprehensive search and cross-checking purposes
- The JBI methodology provides a systematic approach to undertaking and reporting this scoping review
- Gray literature will not be included due to the niche research field and extensive international research collaboration resulting in all available literature being published
- There is no formal quality assessment within a scoping review. A narrative synthesis of the research literature reviewed will be undertaken and published.

For peer review only - http://bmjopen.bmj.com/site/about/guidelines.xhtml

INTRODUCTION

Globally approximately 300,000 children and young people from birth to nineteen years of age are diagnosed with cancer each year[1] and 90,000 of these children die from the disease.[2] Within the United Kingdom (UK) of the 1,420 children under the age of fifteen diagnosed with cancer every year,[3] roughly 250 children will die.[4] Parent involvement in their child's care can include making decisions regarding treatments, administering medications and advocating for their child within the multidisciplinary team. Research has shown that parents wants to be involved in all aspects of their child's care particularly when it comes to making treatment decisions.[5] However, parents are rarely involved in decision-making with initial treatment as this is protocol driven, with reduced choices, but take a more active role if treatments fail and relapse strategies are required.[6]

Studies investigating parental experiences of having a child with cancer have tended to focus on diagnoses such as leukemia or lymphoma, which generally have a good prognosis.[3] Parents of children with a poor-prognosis cancer are under-represented. The needs and experiences of this parent population are likely to be different because: 1) survival rates are lower;[3] 2) the number of children diagnosed with a cancer which has a poor-prognosis is lower;[3] 3) established treatment protocols are not always available due to low numbers of children being diagnosed, resulting in a longer time for clinical trials to become established.

Clinical trials within pediatric oncology have advanced over the past decade resulting in more treatment options for children with a poor-prognosis cancer. There has been a vast number of clinical trials involving immunotherapy and targeted inhibitors which have shown to improve or extend survival and in some cases provide cure within adult cancer.[7-8] As a result, these have been extended into clinical trials for children particularly for those who have a poor-prognosis cancer.[9-10] Reflections on clinical practice have identified where established treatment protocols may not be available, parents subsequently become involved in treatment decision-making. This includes options such as clinical trials and off-protocol treatments. Often parents can make a series of repeated treatment decisions depending on their child's response to treatment and toxicities experienced. This is the case for parents whose child is diagnosed with relapsed neuroblastoma or relapsed Medulloblastoma.

Prognosis relates to the outcome of the cancer diagnosis and whether the disease is likely to return.[11] A poor-prognosis indicates a highly unfavorable treatment outcome likely to result in death. [11] Emerging research conducted by Lichtenthal et al published in 2020 has shown that 73% of parents whose child has a poor-prognosis and subsequently dies experience regret.[12] Regret is predominately related to the decisions parents have made and this regret can result in prolonged grieving.[13]

Research published in 2011 by Tomlinson et al has acknowledged that parents pursue cancer-directed treatments even when the chance of cure is small.[14] Although this research is a decade old, it remains a pertinent piece of research supporting what is seen in clinical practice today. This

For peer review only - http://bmjopen.bmj.com/site/about/guidelines.xhtml

suggests parents continue to pursue cancer-directed treatments regardless of cure outcomes. Quality information including treatment options, risks and benefits, expectations of treatment and cure, to make informed decisions based on parent individual values and preferences is paramount.[5] This is important in treatment decision-making to increase parent empowerment, confidence in the decision made[15] and potentially reduce decisional conflict and regret.[12] Quality information relating to diagnosis, treatment, expectations of cure, potential future impairments, how their child is responding to treatment and how the cancer may have developed are important factors for parents.[16] Parent information needs have highlighted that good quality information results in higher levels of satisfaction in healthcare professionals and the care their child is receiving.[16-17] Research on parent treatment decision-making at the end-of-life or opting to participate in phase I clinical trials has shown parent preferences for cancer-directed treatment includes hoping for a cure, prolong life, decreasing suffering[5,14] and ensure they have done everything possible to save their child's life.[14] With the recognition that parents experience high levels of regret, and want to be involved in treatment decision-making, particularly when their child has a poor-prognosis, this requires an understanding of individual parent values and preferences which inform these decisions.

Values and preferences relate to the expectations, goals, beliefs and predispositions of an individual when making a decision.[18]These values and preferences inform the decision-making process for a person and ultimately define what decision they make. Supporting parents in understanding their own values and preferences and how these inform treatment decision-making is essential to help reduce regret. Support tools to elicit parent values and preferences can help in the process of making decisions.[19]As regret in this parent population is centered on treatment decision-making, understanding parent values and preferences may provide insight into what parents require in terms of support to enable them to make informed decisions.

This scoping review has been prompted by the author's clinical experience of caring for children receiving cancer-directed therapy for a poor-prognosis cancer. Parents are involved in making treatment decisions for their child, often with the need to make a series of repeated treatment decisions as their child's condition changes. This raises questions such as: What are parent values and preferences when their child is receiving cancer-directed therapy for a poor-prognosis cancer? How do these values and preferences inform treatment decision-making?

A preliminary search on MEDLINE using the EBSCO platform in April 2020 found two relevant reviews. A literature review published in 2013 investigated parent decision-making of care and treatment in childhood cancer.[20] This review did not specifically focus on parent values and preferences when their child has a poor-prognosis cancer where decision-making may differ due to the child's condition and likely outcome. It cannot be assumed that values and preferences would be the same for parents regardless of the outcome for their child (survival, death, disabilities as a result of diagnosis/treatment). This warrants specific investigation. A mixed-methods systematic review published in 2014 looked at medico-legal practice in judgements and decisions when a child's

For peer review only - http://bmjopen.bmj.com/site/about/guidelines.xhtml

treatment is not curative.[21] The outcome from this review provides a comprehensive theoretical framework on the processes of parent decision-making. The theoretical framework is of the positive and negative influences in the decision-making process focusing on the transition from cancer-directed therapy to palliative/End-of-Life care for their child.[21] How these decisions relate to individual values and preferences which underpins parents' decision-making, is not included in this framework. The framework also considers the illness trajectory from cancer-directed therapy to palliative/End-of-Life Care to be a linear process, which in clinical practice is not always the case.

This scoping review allows for a primary focus on parent values and preferences when their child is receiving cancer-directed therapy for a poor-prognosis cancer; this has not been specifically addressed previously. This review seeks to map the available research literature of parent values and preferences underpinning treatment decision-making when their child is receiving cancer-directed therapy for a poor-prognosis cancer. Through clarifying these values and preferences, this scoping review will inform further research with parents who are making repeated treatment decisions when their child has a poor-prognosis cancer. Data from this scoping review and data from interviews with parents who are making repeated treatment decisions will be combined., This will inform the development of a decision aid which is underpinned by parent values and preferences to support parents when making treatment decisions. Furthermore, the scoping review may identify research gaps which could be addressed in the future to strengthen the support given to parent when making treatment decisions for their child.

REVIEW OBJECTIVES AND QUESTIONS

The objective is to explore parent values and preferences underpinning treatment decision-making when their child is receiving cancer-directed therapy for a poor-prognosis cancer. This scoping review will seek to answer two questions:

1) What are parent values and preferences when their child is receiving cancer-directed therapy for a poor-prognosis cancer?
2) How do these values and preferences inform treatment decision-making?

INCLUSION CRITERIA

Participants

This will be parents of a child (<18 years) who has a poor-prognosis cancer and is receiving cancer-directed therapy.

Concept

The aim, is to describe, explore and understand the values and preferences which underpin parent treatment decision-making when their child is receiving cancer-directed therapy for a poor-prognosis cancer. The review will specifically focus on studies where the child is receiving cancer-directed therapy, defined as any type of cancer treatment as opposed to end-of-life care, palliative care or symptom management alone. The parent of a child who is receiving cancer-directed therapy and palliative care/symptom management will be included.

For peer review only - http://bmjopen.bmj.com/site/about/guidelines.xhtml

Context

Parents of a child who is receiving cancer-directed therapy for a poor-prognosis cancer in any clinical or medical setting (hospital, community and hospice) will be considered.

Types of evidence sources

International evidence sources for this review will be considered. As this is a small parent population within childhood cancer, international collaboration to enhance knowledge and progress research within this field is well established. Qualitative, quantitative and mixed methods studies drawn from research literature including systematic reviews will be included Literature or systematic reviews which report the same study population as research studies included will be considered eligible as long as different data is presented. Otherwise these articles will be included and used for cross-checking purposes.

Studies included can be prospective or retrospective. Including retrospective studies is important due to the sensitivity of the topic as research has been carried out with parents after their child has died to reduce burden prospectively.

The search will include the last 25 years from 1995 to 2020 inclusive (until the time the database searches take place). This time period has been considered as seminal work was published in 1996 relating to parent treatment decision-making when their child has a first cancer recurrence.[22] In addition, being a small parent population, research can take years to complete due to low participant numbers therefore an extensive search period is required.

METHODS

The Joanna Briggs Institute scoping review methodology will be followed.[23] This scoping review was registered on the Open Science Framework on 2nd June 2020 (https://osf.io/n7j9f). For the scoping review report, the Preferred Reporting Items for Systematic Reviews and Meta-Analyses (PRISMA-ScR) Checklist[24] will be followed (figure 1). The PRISMA flowchart will provide the search process of identification, screening, eligible and included articles for the scoping review (figure 2).

SEARCH STRATEGY

Database searches will ascertain published research studies, literature or systematic reviews. An initial search was completed in Medline and Cumulative Index of Nursing and Allied Health Literature (CINAHL) using the EBSCO platform in April 2020. This initial database search found relevant primary studies using qualitative and quantitative methods and therefore warrants the scoping review being conducted. The Medline search yielded 216 articles and the CINAHL search yielded 115 articles. The key words were devised from known terminology used in clinical practice, academia, definitions (written in the introduction) and literature read on parent decision-making in childhood cancer. Table 1 shows the list of key words and phrases. Each key word was searched for subject headings/MeSH terms to allow for full exploration of the academic language associated with the word. Phrases were not searched for subject headings/MeSH terms as these are natural language phrases

For peer review only - http://bmjopen.bmj.com/site/about/guidelines.xhtml

as opposed to academic language. The Medline search strategy used in the initial database search is detailed in figure 3. From the articles retrieved in this initial search, the text words in the title and abstract were analysed in combination with the index terms used to describe the articles. This did not provide any additional key words or phrases to be included in the database searches.

Table 1: Keywords for Subject Headings/MeSH Terms and Phrases for Database Searches

Keywords searched for Subject Headings/MeSH Terms and Relevant Phrases					
**Parent**	**Value**	**Poor-Prognosis**	**Child**	**Cancer**	**Decision-Making**
Family	Preference	Relapse	Paediatric	Oncology	"decision making"
	Choice	Recurrent	Pediatric	neoplasm	
	Choose	Refractory		Malignancy	
	Belief	Incurable		"Cancer-direct* therap"	
	Attitude	Advanced Cancer			
	Expectation	Uncertainty			
	Predisposition	Deteriorate			
	Influence	Disease progression			
	Experience	"advanc* cancer"			
	Perception	"no realistic chance of cure"			
	"goal* of care"	"disease* progress*"			
		"palliat* chemotherap*"			
		"no reasonable chance of cure"			

For peer rev

The databases that will be searched for this review will be CINAHL, Medline and PsycINFO using the EBSCO platform and the Web of science core collections using the Clarivate platform. The list of key words will be searched for subject headings/MeSH terms separately in each of the databases with the acknowledgement that databases do not have a consistent approach to indexing articles. Phrases will not be searched for subject headings/MeSH terms. The use of search symbols such as truncation, adjacency and the wildcard will allow for full exploration of words. For example, p#ediatric provides inclusion of English and United States spelling of the word. Exact phrase searching with the

For peer review only - http://bmjopen.bmj.com/site/about/guidelines.xhtml

use of quotation marks for example “decision-making” and “no realistic chance of cure” will be incorporated. This allows for inclusion of some commonly used phrases in clinical practice and within the research literature previously read. Boolean operators (AND/OR) will support the combination between key words, subject headings/MeSH terms using OR and the main topics using AND (parent, values and preferences, decision making, poor-prognosis, child, cancer). The main topics combined using AND in the Medline search strategy (figure 3) are Parent (S5), Values and Preferences (S22), Decision Making (S28), Poor-Prognosis (S45), Child (S48) and Cancer (S56).

Inclusion criteria for the review includes: published research studies, literature or systematic reviews written in English with full abstract available from January 1995 onwards. Articles can be from any country. Only articles written in English will be included due to the feasibility of being able to transcribe research published in other languages and the time/financial cost in doing so. Articles will be initially screened on the title and then refined through reading the abstract before retrieving the full-text to confirm inclusion or exclusion. The Web of Science will be used to look at the reference and citation lists of all included full-text articles. This will highlight any additional articles not included in the database searches which may need to be considered for inclusion. Including citation lists is known as snowballing (or pearl growing) and considered an important aspect to ensure inclusivity of all relevant articles.[25] Gray literature will not be included as this is a niche topic with established international collaborations resulting in available research and literature reviews being published. The corresponding author for any article will be contacted if clarification is required for completeness of either including or excluding the article within the scoping review. The database searches will be peer-reviewed by the University Research Librarian for transparency and robustness of the search strategy.

SOURCE OF EVIDENCE SELECTION

Articles obtained will be stored on EndNote X9.2 and duplications removed. Initial screening of the studies will be done by reading the title and abstract to ascertain whether they fit the inclusion criteria. This screening will be carried out independently by two reviewers (HP and ASD) before the review team meet to discuss the articles to be included for full-text review. Any discrepancies will be resolved with the addition of the third reviewer (FG).

Full-text of the articles included based on abstract will be retrieved. These will be read in full by two reviewers (HP and ASD). Corresponding authors of the retrieved articles will be contacted for clarification if required. In the absence of being able to contact the corresponding author of an article, other members of the research team will be approached. The review team will meet again to discuss the final articles to be included in the scoping review and the third reviewer (FG) will resolve any discrepancies. Any full-text articles which are excluded at this stage will be justified in the scoping review report. For transparency the search results will be presented in the PRISMA flowchart (figure 2) which will systematically detail the selection process of articles.

For peer review only - http://bmjopen.bmj.com/site/about/guidelines.xhtml
For peer review only

DATA EXTRACTION

Data will be extracted from the articles obtained which meet the objectives of the scoping review. Data extraction will be undertaken in full by two reviewers (HP and ASD) and the third reviewer (FG) will randomly select 25% of the eligible articles to review. The third reviewer (FG) will review data extraction templates completed by the two full reviewers (HP and ASD) against the relevant article. This is a quality check to ensure the extracted data from the articles is accurate and is executed with rigour. Once the data extraction and review of the randomly selection articles has been completed independently by all three reviewers, the review team will meet to discuss and address any discrepancies.

For a consistent approach between reviewers, the Joanna Briggs Institute source of evidence template[23] will be modified to extract information, characteristics and findings from the articles which are relevant to answering the scoping review questions. This will include information on the population, concept, context, type of evidence source, study methods, values and preferences, information on what cancer-directed therapy was given and whether this was in conjunction with palliative care/symptom management. The modified data extraction template for this scoping review can be found in figure 2. Any amendments to the extraction template during the scoping review will be highlighted in the scoping review report.

ANALYSIS OF THE EVIDENCE

Analysis will commence with the final number of articles included in the scoping review and the basic characteristics of these articles. The data extraction template (figure 4) will highlight the most consistent and clear way for the evidence to be presented which is representative of the extracted data once the scoping review is complete. The data extraction template will record basic characteristics of the articles' such as first author, year of publication, country, study design (ie: qualitative, quantitative, mixed-methods, retrospective, prospective, longitudinal, cohort), research methods, and the number of participants in each study. This information will not be analysed but will be presented in the scoping review report to provide an overview of the included literature.

For qualitative data, content analysis will provide a description of data extracted and will be mapped to show consistencies and differences amongst the data.

For quantitative data, the frequency and numerical information will be presented based on the data extracted. For example, the sample size and types of study design could be individually displayed in graph or tabular format. In both the qualitative and quantitative data extraction, the analysis will reflect the objectives for the scoping review.

PRESENTATION OF RESULTS

Results will be presented in formats such as tabular, diagrammatic and descriptive summaries to clearly represent the extracted data relevant to the scoping review objectives. The basic characteristics of the studies will be presented in table format. This will include the citation, country, study design, methods and sample size of each article. This will provide the reader with an overall summary of the studies which are included in the scoping review. Descriptive summaries of the

For peer review only - http://bmjopen.bmj.com/site/about/guidelines.xhtml

qualitative data will be supported with visual diagrams mapping the relevant categories of the scoping review objectives. For example, a word diagram to illustrate the frequency of values and preferences which come from the data and subsequently mapping these concepts into themes to show inter-dependent relationships between these values and preferences. Quantitative data will be presented in graphs and figures to show comparisons and differences between the data extracted. The presentation of results may be refined during the scoping review to align with the data extracted from the included articles. Amendments to the suggested presentation will be highlighted in the scoping review report.

The scoping review will identify any research gaps which could enhance how support is provided to this parent population. Recommendations for practice which may support parents when making treatment decisions for cancer-directed therapy when their child has a poor-prognosis cancer will be highlighted.

ETHICS AND DISSEMINATION:

This scoping review is the first to look at parent values and preferences in treatment decision-making when their child has a poor-prognosis cancer. The results from this scoping review will be used to inform an interview schedule with parents to understand their values and preferences when making repeated treatment decisions for their child with relapse neuroblastoma, a poor prognosis childhood cancer. The combined results from this scoping review and parent interviews will inform the development of a decision aid to support parents in their treatment decision-making. This data and the decision aid developed will be published in peer-reviewed journals and presented at national/international conferences consisting of healthcare professionals, charities and parents. This scoping review does not require ethical approval.

For peer review only

For peer review only - http://bmjopen.bmj.com/site/about/guidelines.xhtml

REFERENCES

1. Steliarova-Foucher E, Colombet M, Ries LAG, et al. International incidence of childhood cancer, 2001-10: a population-based registry study. *Lancet Oncol* 2017;18(6):719-731. doi: 10.1016/S1470-2045(17)30186-9 [published Online first: 11 April 2017].
2. Childhood Cancer International. [Internet] 2020 [cited 2020 May 30]. Available from: https://www.childhoodcancerinternational.org/
3. Public Health England. Childhood Cancer Statistics, England Annual Report 2018. [Internet] 2018 [cited 2020 Apr 28]. Available from: http://www.ncin.org.uk/publications/reports
4. Cancer Research UK. Children's cancer statistics. [Internet] 2019 [cited 2020 Apr 28]. Available from:https://www.cancerresearchuk.org/health-professional/cancer-statistics/childrens-cancers
5. Mack JW, Feudtner C, Hinds PS. Communication and decision support for children with advanced cancer and their families. *Am Soc Clin Oncol Educ Book*. 2012;32:637-43. doi: 10.14694/EdBook_AM.2012.32.164. [published Online first: 17 May, 2012].
6. Hinds PS, Oakes L, Furman W, et al. Decision making by parents and healthcare professionals when considering continued care for pediatric patients with cancer. *Oncol Nurs Forum* 1997;24(9):1523-28.
7. Lynch A, Murphy A. The emerging role of immunotherapy in colorectal cancer. *Ann Transl Med* 2016;4(16). doi: 10.21037/atm.2016.08.29.
8. Somasundaram A, Burns TF. The next generation of immunotherapy: keeping lung cancer in check. *J Hematol Oncol* 2017;10(1):87. doi: 10.1186/s13045-017-0456-5.
9. Mackall CL, Merchant MS, Fry TJ. Immune-based therapies for childhood cancer. *Nat Rev Clin Oncol* 2014;11:693–703. doi: 10.1186/s13045-017-0456-5. [published Online first: 28 October 2014].
10. Roberts SS, Chou AJ, Cheung N-KV. Immunotherapy of childhood sarcomas. *Front Oncol* 2015;5:181. doi: 10.3389/fonc.2015.00181.
11. National Cancer Institute. Understanding Cancer Prognosis. [Internet] 2019 [cited 2020 Mar 31]. Avaliable from: https://www.cancer.gov/about-cancer/diagnosis-staging/prognosis
12. Lichtenthal WG, Roberts KE, Catarozoli C, et al. Regret and unfinished business in parents bereaved by cancer: A mixed methods study. *J Palliat Med* 2020;34(3):367-377. doi: 10.1177/0269216319900301. [published Online first: 5 February 2020].
13. Lannen PK, Wolfe K, Prigerson HG, et al. Unresolved grief in a national sample of bereaved parents: impaired mental and physical health 4 to 9 years later. *J Clin Oncol* 2008;26(36):5870-5876. doi: 10.1200/JCO.2007.14.6738. [published Online first: 24 November 2008].
14. Tomlinson D, Bartels U, Gammon J, et al. Chemotherapy versus supportive care alone in pediatric palliative care for cancer: comparing the preferences of parents and healthcare professionals. *Can Med Assoc J* 2011;183(7):E1252-58. doi: 10.1503/cmaj.110392. [published Online first: 17 October 2011].

For peer review only - http://bmjopen.bmj.com/site/about/guidelines.xhtml

15. Robertson EG, Wakefield CE, Shaw J, et al. Decision-making in childhood cancer: parents' and adolescents' views and perceptions. *Support Care Cancer* 2019;27:4331-4340. doi: 10.1007/s00520-019-04728-x.
16. Kaye E, Mack JW. Parent perceptions of the quality of information received about a child's cancer. *Pediatr Blood Cancer* 2013;60:1896-1901. doi: 10.1002/pbc.24652.
17. Kilicarslan-Toruner E, Akgun-Citak E. Information-seeking behaviours and decision-making process of parents of children with cancer. *Eur J Oncol Nurs* 2013;17(2):176-183. doi: 10.1016/j.ejon.2012.03.001.
18. Straus SE, Richardson WS, Glasziou P, et al. Evidence-Based Medicine How to Practice and Teach EBM. 3rd ed. Edinburgh: Elsevier Churchill Livingstone 2005:12.
19. Marron JM, Mack JW. When to say when: How aggressively to care for children with multiply relapsed cancer?. *Pediatr Blood Cancer* 2015;62(7):1119-20. doi: 10.1002/pbc.25476. [published Online first: 8 March 2015].
20. Markward MJ, Benner K, Freese R. Perspectives of parents on making decisions about the care and treatment of a child with cancer: A review of the literature. *Fam Syst Health* 2013;31(4):406-413. doi: 10.1037/a0034440.
21. Valdez-Martinez E, Noyes J, Bedolla M. When to stop? Decision-making when children's cancer treatment is no longer curative: a mixed methods systematic review. *BMC Pediatr* 2014;4(24):124. doi: 10.1186/1471-2431-14-124.
22. Hinds PS, Birenbaum LK, Clarke-Steffen L. Coming to terms: Parents' response to a first cancer recurrence in their child. *Nurs Res* 1996;45(3):148-153. Doi: 10.1097/00006199-199605000-00005.
23. Peters MDJ, Godfrey C, McInerney P, et al. Chapter 11: Scoping Reviews (2020 version). In: Aromataris E, Munn Z. (Editors) Joanna Briggs Institute Reviewer's Manual, JBI, 2020. Available from: https://reviewersmanual.joannabriggs.org/
24. Tricco, AC, Lillie E, Zarin W, et al. PRISMA Extension for Scoping Reviews (PRISMA-ScR): Checklist and Explanation. *Ann Intern Med* 2018;169(7): 467-73. doi: 10.7326/M18-0850. [published Online first: 4 September 2018].
25. Greenhalgn T, Peacock R. Effectiveness and efficiency of search methods in systematic reviews of complex evidence: audit of primary sources. *BMJ* 2005; 331:1064-1065. doi: 10.1136/bmj.38636.593461.68. [published Online first: 17 October 2005].

PATIENT AND PUBLIC INVOLVEMENT

No patient involvement for this scoping review.

AUTHOR CONTRIBUTIONS

HP, FG, ASD conceived the idea for this scoping review. HP developed the research questions, objectives, inclusion criteria, undertook initial database search, drafted and edited this manuscript. FG and ASD reviewed inclusion criteria, screened abstracts and full-text papers from initial database search, and edited this manuscript. HP, FG, ASD approved final manuscript version.

For peer review only

FUNDING STATEMENT:

Helen Pearson is supported by National Institute Health Research (NIHR) Clinical Doctoral Research Fellowship NIHR300548.

ACKNOWLEDGEMENTS:

This research was supported in part by the NIHR Great Ormond Street Hospital Biomedical Research Centre.

COMPETING INTEREST STATEMENT

None declared

FIGURE LEGENDS

Figure 1: PRISMA ScR Checklist

Figure 2: PRISMA Flowchart

Figure 3: Medline Search Strategy

Figure 2: JBI Data Extraction Tool

For peer review only

Figure 1 Preferred Reporting Items for Systematic reviews and Meta-Analyses extension for Scoping Reviews (PRISMA-ScR) Checklist

SECTION	ITEM	PRISMA-ScR CHECKLIST ITEM	REPORTED ON PAGE #
**TITLE**			
Title	1	Identify the report as a scoping review.	Click here to enter text.
**ABSTRACT**			
Structured summary	2	Provide a structured summary that includes (as applicable): background, objectives, eligibility criteria, sources of evidence, charting methods, results, and conclusions that relate to the review questions and objectives.	Click here to enter text.
**INTRODUCTION**			
Rationale	3	Describe the rationale for the review in the context of what is already known. Explain why the review questions/objectives lend themselves to a scoping review approach.	Click here to enter text.
Objectives	4	Provide an explicit statement of the questions and objectives being addressed with reference to their key elements (e.g., population or participants, concepts, and context) or other relevant key elements used to conceptualize the review questions and/or objectives.	Click here to enter text.
**METHODS**			
Protocol and registration	5	Indicate whether a review protocol exists; state if and where it can be accessed (e.g., a Web address); and if available, provide registration information, including the registration number.	Click here to enter text.
Eligibility criteria	6	Specify characteristics of the sources of evidence used as eligibility criteria (e.g., years considered, language, and publication status), and provide a rationale.	Click here to enter text.
Information sources*	7	Describe all information sources in the search (e.g., databases with dates of coverage and contact with authors to identify additional sources), as well as the date the most recent search was executed.	Click here to enter text.
Search	8	Present the full electronic search strategy for at least 1 database, including any limits used, such that it could be repeated.	Click here to enter text.
Selection of sources of evidence†	9	State the process for selecting sources of evidence (i.e., screening and eligibility) included in the scoping review.	Click here to enter text.
Data charting process‡	10	Describe the methods of charting data from the included sources of evidence (e.g., calibrated forms or forms that have been tested by the team before their use, and whether data charting was done independently or in duplicate) and any processes for obtaining and confirming data from investigators.	Click here to enter text.
Data items	11	List and define all variables for which data were sought and any assumptions and simplifications made.	Click here to enter text.
Critical appraisal of individual sources of evidence§	12	If done, provide a rationale for conducting a critical appraisal of included sources of evidence; describe the methods used and how this information was used in any data synthesis (if appropriate).	Click here to enter text.

For peer review only

SECTION	ITEM	PRISMA-ScR CHECKLIST ITEM	REPORTED ON PAGE #
Synthesis of results	13	Describe the methods of handling and summarizing the data that were charted.	Click here to enter text.
**RESULTS**			
Selection of sources of evidence	14	Give numbers of sources of evidence screened, assessed for eligibility, and included in the review, with reasons for exclusions at each stage, ideally using a flow diagram.	Click here to enter text.
Characteristics of sources of evidence	15	For each source of evidence, present characteristics for which data were charted and provide the citations.	Click here to enter text.
Critical appraisal within sources of evidence	16	If done, present data on critical appraisal of included sources of evidence (see item 12).	Click here to enter text.
Results of individual sources of evidence	17	For each included source of evidence, present the relevant data that were charted that relate to the review questions and objectives.	Click here to enter text.
Synthesis of results	18	Summarize and/or present the charting results as they relate to the review questions and objectives.	Click here to enter text.
**DISCUSSION**			
Summary of evidence	19	Summarize the main results (including an overview of concepts, themes, and types of evidence available), link to the review questions and objectives, and consider the relevance to key groups.	Click here to enter text.
Limitations	20	Discuss the limitations of the scoping review process.	Click here to enter text.
Conclusions	21	Provide a general interpretation of the results with respect to the review questions and objectives, as well as potential implications and/or next steps.	Click here to enter text.
**FUNDING**			
Funding	22	Describe sources of funding for the included sources of evidence, as well as sources of funding for the scoping review. Describe the role of the funders of the scoping review.	Click here to enter text.

JBI = Joanna Briggs Institute; PRISMA-ScR = Preferred Reporting Items for Systematic reviews and Meta-Analyses extension for Scoping Reviews.
* Where *sources of evidence* (see second footnote) are compiled from, such as bibliographic databases, social media platforms, and Web sites.
† A more inclusive/heterogeneous term used to account for the different types of evidence or data sources (e.g., quantitative and/or qualitative research, expert opinion, and policy documents) that may be eligible in a scoping review as opposed to only studies. This is not to be confused with *information sources* (see first footnote).
‡ The frameworks by Arksey and O'Malley (6) and Levac and colleagues (7) and the JBI guidance (4, 5) refer to the process of data extraction in a scoping review as data charting.
§ The process of systematically examining research evidence to assess its validity, results, and relevance before using it to inform a decision. This term is used for items 12 and 19 instead of "risk of bias" (which is more applicable to systematic reviews of interventions) to include and acknowledge the various sources of evidence that may be used in a scoping review (e.g., quantitative and/or qualitative research, expert opinion, and policy document).

For peer review only

Figure 2. Preferred Reporting Items for Systematic Reviews and Meta-Analyses (PRISMA) flowchart for the scoping review

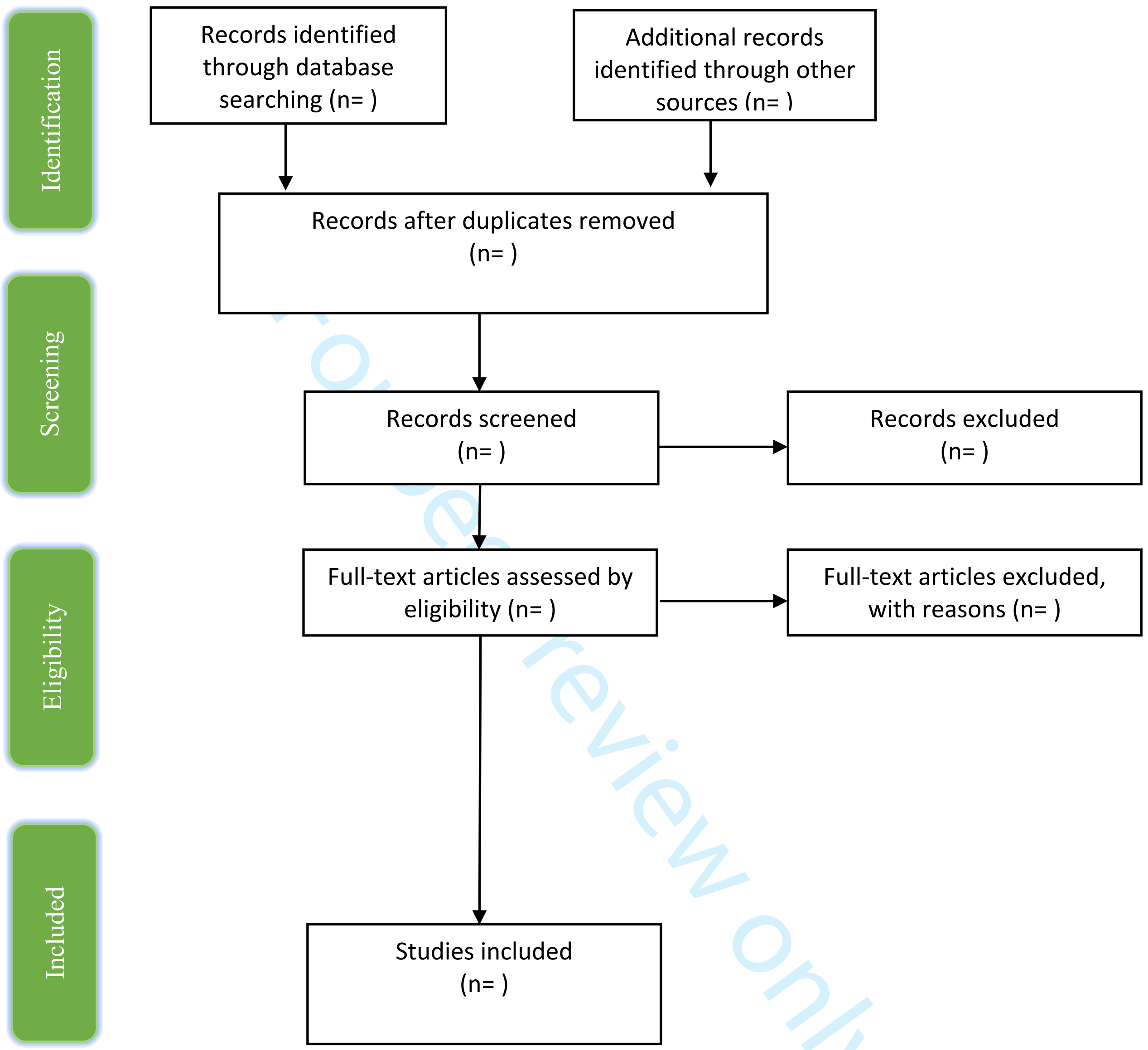

Figure 3: Medline Search Strategy

Medline Search Strategy
S1 parent*
S2 mother*
S3 father*
S4 famil*
S5 S1 OR S2 OR S3 OR S4
S6 value*
S7 (MH "Value of Life") OR (MH "Social Values")
S8 preference*
S9 "goal* of care"
S10 choice*
S11 belief*
S12 (MH "Social Norms")
S13 attitude*
S14 expect*
S15 (MH "Motivation")
S16 predisposition*
S17 influenc*
S18 experienc*
S19 (MH "Clinical Decision-Making")
S20 choose*
S21 percept*
S22 S6 OR S7 OR S8 OR S9 OR S10 OR S11 OR S12 OR S13 OR S14 OR S15 OR S16 OR S17 OR S18 OR S19 OR S20 OR S21

For peer review only

S23 "decision making"
S24 (MH "Decision Making, Shared")
S25 decision N2 making
S26 decision*
S27 decide*
S28 S23 OR S24 OR S25 OR S26 OR S27
S29 S5 AND S22 AND S28
S30 "poor prognosis"
S31 relaps*
S32 (MH "Recurrence")
S33 recurrenc*
S34 refractory
S35 incurable
S36 (MH "Terminally Ill")
S37 "advanc* cancer"
S38 "no realistic chance of cure"
S39 uncertain*
S40 deteriorat*
S41 (MH "Clinical Deterioration")
S42 "disease* progress*"
S43 "palliat* chemotherap*"
S44 "no reasonable chance of cure"
S45 S30 OR S31 OR S32 OR S33 OR S34 OR S35 OR S36 OR S37 OR S38 OR S39 OR S40 OR S41 OR S42 OR S43 OR S44
S46 child*

For peer review only

S47	p#ediatric*
S48	S46 OR S47
S49	cancer
S50	neoplasm*
S51	(MH "Neoplasm Recurrence, Local")
S52	malignan*
S53	(MH "Neoplasms")
S54	"cancer direct* therap*"
S55	oncolog*
S56	S49 OR S50 OR S51 OR S52 OR S53 OR S54 OR S55
S57	S45 AND S48 AND S56
S58	S29 AND S57

For peer review only

Figure 4: JBI Data Extraction Template amended for this Scoping Review

Scoping Review Details	
Scoping Review Title	
Review Objectives	
Review Questions	
Inclusion/Exclusion Criteria	
Population (parent of a child under age of 18 years)	
Concept (parent of a child receiving cancer-directed therapy for a poor prognosis cancer)	
Context (hospital, community, hospice)	
Types of Evidence Source (Qual/Quant, mixed methods)	
Evidence source Details and Characteristics	
Citation details (author, year of publication, journal)	
Country	
Study Aims/Objectives	
Prospective or Retrospective study	

For peer review only

Longitudinal or Cohort study	
Research Methodology & Methods	
Participant Details (mother,father sample size)	
Data extracted from sources of evidence	
Cancer-directed therapy options available (list)	
Cancer-directed therapy in conjunction with palliative care/symptoms management	YES / NO
Cancer-directed therapy given to child (list if more than one)	
Outcome from cancer-directed therapy given	
Values and preferences identified (list)	
Explanation of how these values and preferences informed decision-making (explicitly stated/not stated/unsure)	
Article recommendations for future research/clinical practice	

For peer review only

Parent values and preferences underpinning treatment decision-making in poor-prognosis childhood cancer: A scoping review protocol

Journal:	*BMJ Open*
Manuscript ID	bmjopen-2020-046284.R2
Article Type:	Protocol
Date Submitted by the Author:	20-Apr-2021
Complete List of Authors:	Pearson, Helen; Royal Marsden NHS Foundation Trust, ; University of Southampton Faculty of Medicine Health and Life Sciences,<br>Gibson, Faith; University of Surrey, Faculty of Health and Medical Sciences; Great Ormond Street Hospital For Children NHS Foundation Trust,<br>Darlington, Anne-Sophie; University of Southamptom, Faculty of Health Sciences
**Primary Subject Heading**:	Paediatrics
Secondary Subject Heading:	Oncology
Keywords:	Paediatric oncology < PAEDIATRICS, Paediatric palliative care < PALLIATIVE CARE, Cancer pain < ONCOLOGY

I, the Submitting Author has the right to grant and does grant on behalf of all authors of the Work (as defined in the below author licence), an exclusive licence and/or a non-exclusive licence for contributions from authors who are: i) UK Crown employees; ii) where BMJ has agreed a CC-BY licence shall apply, and/or iii) in accordance with the terms applicable for US Federal Government officers or employees acting as part of their official duties; on a worldwide, perpetual, irrevocable, royalty-free basis to BMJ Publishing Group Ltd ("BMJ") its licensees and where the relevant Journal is co-owned by BMJ to the co-owners of the Journal, to publish the Work in this journal and any other BMJ products and to exploit all rights, as set out in our licence.

The Submitting Author accepts and understands that any supply made under these terms is made by BMJ to the Submitting Author unless you are acting as an employee on behalf of your employer or a postgraduate student of an affiliated institution which is paying any applicable article publishing charge ("APC") for Open Access articles. Where the Submitting Author wishes to make the Work available on an Open Access basis (and intends to pay the relevant APC), the terms of reuse of such Open Access shall be governed by a Creative Commons licence – details of these licences and which Creative Commons licence will apply to this Work are set out in our licence referred to above.

Other than as permitted in any relevant BMJ Author's Self Archiving Policies, I confirm this Work has not been accepted for publication elsewhere, is not being considered for publication elsewhere and does not duplicate material already published. I confirm all authors consent to publication of this Work and authorise the granting of this licence.

For peer review only

Parent values and preferences underpinning treatment decision-making in poor-prognosis childhood cancer: A scoping review protocol

Helen Pearson[1,2] Professor Faith Gibson[3,4] Professor Anne-Sophie Darlington[1]

1. School of Health Sciences, University of Southampton, Southampton, United Kingdom
2. The Oak Centre for Children and Young People, The Royal Marsden NHS Foundation Trust, Sutton, Surrey, United Kingdom
3. Centre for Outcomes and Experience Research in Children's Health, Illness and Disability (ORCHID), Great Ormond Street Hospital for Children NHS Foundation Trust, London, United Kingdom
4. School of Health Sciences, University of Surrey, Guildford, Surrey, United Kingdom

Corresponding Author:
Helen Pearson
Helenpearson1@nhs.net

Word count: 2830

Keywords:
Cancer; Child; Decision-making; Parent; Poor-Prognosis

For peer review only - http://bmjopen.bmj.com/site/about/guidelines.xhtml

ABSTRACT

Introduction:

Parents of a child with cancer want to be involved in making treatment decisions for their child. Underpinning and informing these decisions are parents' individual values and preferences. Parents of a child who has a poor-prognosis cancer and who subsequently dies can experience decisional regret. To support parents, and potentially reduce decisional regret, identifying the values and preferences of parents who are making these treatment decisions may enhance the support which can be provided by healthcare professionals. An increased understanding will support future work in this area and identify research gaps which could strengthen support strategies in clinical practice. The aim of this scoping review is to explore parent values and preferences underpinning treatment decision-making when their child is receiving cancer-directed therapy for a poor-prognosis cancer.

Methods and Analysis:

The JBI scoping review methodology will be followed. An initial database search of CINHAL and Medline will be conducted to analyze the keywords using subject headings and MeSH terms. Articles will be initially screened on title and abstract. The reference and citation lists of the full-text articles to be included will be searched using Web of Science. Articles will be independently reviewed by two reviewers and any discrepancies discussed with a third reviewer. Data extracted will be presented in tabular, diagrams and descriptive summaries.

Ethics and Dissemination:

Ethical approval is not required for this scoping review. This review will inform further research with parents to understand their values and preferences when making repeated treatment decisions when their child has a poor-prognosis cancer. All outputs will be disseminated through peer-reviewed publications and conference presentations.

This scoping review is registered on the Open Science Framework (https://osf.io/n7j9f).

STRENGHS AND LIMTITATIONS OF THIS STUDY

- This is the first scoping review to identify parent values and preferences in treatment decision-making when their child has a poor-prognosis cancer
- The search strategy includes the reference and citation lists of all included full-text articles for a comprehensive search and cross-checking purposes
- The JBI methodology provides a systematic approach to undertaking and reporting this scoping review
- Gray literature will not be included due to the niche research field and extensive international research collaboration resulting in all available literature being published
- There is no formal quality assessment within a scoping review. A narrative synthesis of the research literature reviewed will be undertaken and published.

For peer review only - http://bmjopen.bmj.com/site/about/guidelines.xhtml

INTRODUCTION

Globally approximately 300,000 children and young people from birth to nineteen years of age are diagnosed with cancer each year[1] and 90,000 of these children die from the disease.[2] Within the United Kingdom (UK) of the 1,420 children under the age of fifteen diagnosed with cancer every year,[3] roughly 250 children will die.[4] Parent involvement in their child's care can include making decisions regarding treatments, administering medications and advocating for their child within the multidisciplinary team. Research has shown that parents wants to be involved in all aspects of their child's care particularly when it comes to making treatment decisions.[5] However, parents are rarely involved in decision-making with initial treatment as this is protocol driven, with reduced choices, but take a more active role if treatments fail and relapse strategies are required.[6]

Studies investigating parental experiences of having a child with cancer have tended to focus on diagnoses such as leukemia or lymphoma, which generally have a good prognosis.[3] Parents of children with a poor-prognosis cancer are under-represented. The needs and experiences of this parent population are likely to be different because: 1) survival rates are lower;[3] 2) the number of children diagnosed with a cancer which has a poor-prognosis is lower;[3] 3) established treatment protocols are not always available due to low numbers of children being diagnosed, resulting in a longer time for clinical trials to become established.

Clinical trials within pediatric oncology have advanced over the past decade resulting in more treatment options for children with a poor-prognosis cancer. There has been a vast number of clinical trials involving immunotherapy and targeted inhibitors which have shown to improve or extend survival and in some cases provide cure within adult cancer.[7-8] As a result, these have been extended into clinical trials for children particularly for those who have a poor-prognosis cancer.[9-10] Reflections on clinical practice have identified where established treatment protocols may not be available, parents subsequently become involved in treatment decision-making. This includes options such as clinical trials and off-protocol treatments. Often parents can make a series of repeated treatment decisions depending on their child's response to treatment and toxicities experienced. This is the case for parents whose child is diagnosed with relapsed neuroblastoma or relapsed Medulloblastoma.

Prognosis relates to the outcome of the cancer diagnosis and whether the disease is likely to return.[11] A poor-prognosis indicates a highly unfavorable treatment outcome likely to result in death. [11] Emerging research conducted by Lichtenthal et al published in 2020 has shown that 73% of parents whose child has a poor-prognosis and subsequently dies experience regret.[12] Regret is predominately related to the decisions parents have made and this regret can result in prolonged grieving.[13]

Research published in 2011 by Tomlinson et al has acknowledged that parents pursue cancer-directed treatments even when the chance of cure is small.[14] Although this research is a decade old, it remains a pertinent piece of research supporting what is seen in clinical practice today. This

For peer review only - http://bmjopen.bmj.com/site/about/guidelines.xhtml

suggests parents continue to pursue cancer-directed treatments regardless of cure outcomes. Quality information including treatment options, risks and benefits, expectations of treatment and cure, to make informed decisions based on parent individual values and preferences is paramount.[5] This is important in treatment decision-making to increase parent empowerment, confidence in the decision made[15] and potentially reduce decisional conflict and regret.[12] Quality information relating to diagnosis, treatment, expectations of cure, potential future impairments, how their child is responding to treatment and how the cancer may have developed are important factors for parents.[16] Research investigating parent information needs has highlighted that good quality information results in higher levels of satisfaction in healthcare professionals and the care their child is receiving.[16-17] Research on parent treatment decision-making at the end-of-life or opting to participate in phase I clinical trials has shown parent preferences for cancer-directed treatment includes hoping for a cure, prolong life, decreasing suffering[5,14] and ensure they have done everything possible to save their child's life.[14] With the recognition that parents experience high levels of regret, and want to be involved in treatment decision-making, particularly when their child has a poor-prognosis, this requires an understanding of individual parent values and preferences which inform these decisions.

Values and preferences relate to the expectations, goals, beliefs and predispositions of an individual when making a decision.[18]These values and preferences inform the decision-making process for a person and ultimately define what decision they make. Supporting parents in understanding their own values and preferences and how these inform treatment decision-making is essential to help reduce regret. Support tools to elicit parent values and preferences can help in the process of making decisions.[19]As regret in this parent population is centered on treatment decision-making, understanding parent values and preferences may provide insight into what parents require in terms of support to enable them to make informed decisions.

This scoping review has been prompted by the author's clinical experience of caring for children receiving cancer-directed therapy for a poor-prognosis cancer. Parents are involved in making treatment decisions for their child, often with the need to make a series of repeated treatment decisions as their child's condition changes. This raises questions such as: What are parent values and preferences when their child is receiving cancer-directed therapy for a poor-prognosis cancer? How do these values and preferences inform treatment decision-making?

A preliminary search on MEDLINE using the EBSCO platform in April 2020 found two relevant reviews. A literature review published in 2013 investigated parent decision-making of care and treatment in childhood cancer.[20] This review did not specifically focus on parent values and preferences when their child has a poor-prognosis cancer where decision-making may differ due to the child's condition and likely outcome. It cannot be assumed that values and preferences would be the same for parents regardless of the outcome for their child (survival, death, disabilities as a result of diagnosis/treatment). This warrants specific investigation. A mixed-methods systematic review published in 2014 looked at medico-legal practice in judgements and decisions when a child's

For peer review only - http://bmjopen.bmj.com/site/about/guidelines.xhtml

treatment is not curative.[21] The outcome from this review provides a comprehensive theoretical framework on the processes of parent decision-making. The theoretical framework is of the positive and negative influences in the decision-making process focusing on the transition from cancer-directed therapy to palliative/End-of-Life care for their child.[21] How these decisions relate to individual values and preferences which underpins parents' decision-making, is not included in this framework. The framework also considers the illness trajectory from cancer-directed therapy to palliative/End-of-Life Care to be a linear process, which in clinical practice is not always the case.

This scoping review allows for a primary focus on parent values and preferences when their child is receiving cancer-directed therapy for a poor-prognosis cancer; this has not been specifically addressed previously. This review seeks to map the available research literature of parent values and preferences underpinning treatment decision-making when their child is receiving cancer-directed therapy for a poor-prognosis cancer. Through clarifying these values and preferences, this scoping review will inform further research with parents who are making repeated treatment decisions when their child has a poor-prognosis cancer. Data from this scoping review and data from interviews with parents who are making repeated treatment decisions will be combined., This will inform the development of a decision aid which is underpinned by parent values and preferences to support parents when making treatment decisions. Furthermore, the scoping review may identify research gaps which could be addressed in the future to strengthen the support given to parent when making treatment decisions for their child.

REVIEW OBJECTIVES AND QUESTIONS

The objective is to explore parent values and preferences underpinning treatment decision-making when their child is receiving cancer-directed therapy for a poor-prognosis cancer. This scoping review will seek to answer two questions:

1) What are parent values and preferences when their child is receiving cancer-directed therapy for a poor-prognosis cancer?
2) How do these values and preferences inform treatment decision-making?

INCLUSION CRITERIA

Participants

This will be parents of a child (<18 years) who has a poor-prognosis cancer and is receiving cancer-directed therapy.

Concept

The aim, is to describe, explore and understand the values and preferences which underpin parent treatment decision-making when their child is receiving cancer-directed therapy for a poor-prognosis cancer. The review will specifically focus on studies where the child is receiving cancer-directed therapy, defined as any type of cancer treatment as opposed to end-of-life care, palliative care or symptom management alone. The parent of a child who is receiving cancer-directed therapy and palliative care/symptom management will be included.

For peer review only - http://bmjopen.bmj.com/site/about/guidelines.xhtml

Context

Parents of a child who is receiving cancer-directed therapy for a poor-prognosis cancer in any clinical or medical setting (hospital, community and hospice) will be considered.

Types of evidence sources

International evidence sources for this review will be considered. As this is a small parent population within childhood cancer, international collaboration to enhance knowledge and progress research within this field is well established. Qualitative, quantitative and mixed methods studies drawn from research literature including systematic reviews will be included Literature or systematic reviews which report the same study population as research studies included will be considered eligible as long as different data is presented. Otherwise these articles will be included and used for cross-checking purposes.

Studies included can be prospective or retrospective. Including retrospective studies is important due to the sensitivity of the topic as research has been carried out with parents after their child has died to reduce burden prospectively.

The search will include the last 25 years from 1995 to 2020 inclusive (until the time the database searches take place). This time period has been considered as seminal work was published in 1996 relating to parent treatment decision-making when their child has a first cancer recurrence.[22] In addition, being a small parent population, research can take years to complete due to low participant numbers therefore an extensive search period is required.

METHODS

The Joanna Briggs Institute scoping review methodology will be followed.[23] This scoping review was registered on the Open Science Framework on 2nd June 2020 (https://osf.io/n7j9f). For the scoping review report, the Preferred Reporting Items for Systematic Reviews and Meta-Analyses (PRISMA-ScR) Checklist[24] will be followed (see online supplementary figure 1). The PRISMA flowchart will provide the search process of identification, screening, eligible and included articles for the scoping review (figure 1).

SEARCH STRATEGY

Database searches will ascertain published research studies, literature or systematic reviews. An initial search was completed in Medline and Cumulative Index of Nursing and Allied Health Literature (CINAHL) using the EBSCO platform in April 2020. This initial database search found relevant primary studies using qualitative and quantitative methods and therefore warrants the scoping review being conducted. The Medline search yielded 216 articles and the CINAHL search yielded 115 articles. The key words were devised from known terminology used in clinical practice, academia, definitions (written in the introduction) and literature read on parent decision-making in childhood cancer. Table 1 shows the list of key words and phrases. Each key word was searched for subject headings/MeSH terms to allow for full exploration of the academic language associated with the word.

For peer review only - http://bmjopen.bmj.com/site/about/guidelines.xhtml

Phrases were not searched for subject headings/MeSH terms as these are natural language phrases as opposed to academic language. The Medline search strategy used in the initial database search is detailed in figure 2, 3 and 4. From the articles retrieved in this initial search, the text words in the title and abstract were analysed in combination with the index terms used to describe the articles. This did not provide any additional key words or phrases to be included in the database searches.

Table 1: Keywords for Subject Headings/MeSH Terms and Phrases for Database Searches

Keywords searched for Subject Headings/MeSH Terms and Relevant Phrases					
**Parent**	**Value**	**Poor-Prognosis**	**Child**	**Cancer**	**Decision-Making**
Family	Preference	Relapse	Paediatric	Oncology	"decision making"
	Choice	Recurrent	Pediatric	neoplasm	
	Choose	Refractory		Malignancy	
	Belief	Incurable		"Cancer-direct* therap"	
	Attitude	Advanced Cancer			
	Expectation	Uncertainty			
	Predisposition	Deteriorate			
	Influence	Disease progression			
	Experience	"advanc* cancer"			
	Perception	"no realistic chance of cure"			
	"goal* of care"	"disease* progress*"			
		"palliat* chemotherap*"			
		"no reasonable chance of cure"			

The databases to be searched for this review will be CINAHL, Medline and PsycINFO using the EBSCO platform and the Web of science core collections using the Clarivate platform. The list of key words will be searched for subject headings/MeSH terms separately in each of the databases with the acknowledgement that databases do not have a consistent approach to indexing articles. Phrases will not be searched for subject headings/MeSH terms. The use of search symbols such as truncation, adjacency and the wildcard will allow for full exploration of words. For example, p#ediatric

For peer review only - http://bmjopen.bmj.com/site/about/guidelines.xhtml

provides inclusion of English and United States spelling of the word. Exact phrase searching with the use of quotation marks for example “decision-making” and “no realistic chance of cure” will be incorporated. This allows for inclusion of some commonly used phrases in clinical practice and within the research literature previously read. Boolean operators (AND/OR) will support the combination between key words, subject headings/MeSH terms using OR and the main topics using AND (parent, values and preferences, decision making, poor-prognosis, child, cancer). The main topics combined using AND in the Medline search strategy (figure 2, 3 and 4) are Parent (S5), Values and Preferences (S22), Decision Making (S28), Poor-Prognosis (S45), Child (S48) and Cancer (S56).

Inclusion criteria for the review includes: published research studies, literature or systematic reviews written in English with full abstract available from January 1995 onwards. Articles can be from any country. Only articles written in English will be included due to the feasibility of being able to transcribe research published in other languages and the time/financial cost in doing so. Articles will be initially screened on the title and then refined through reading the abstract before retrieving the full-text to confirm inclusion or exclusion. The Web of Science will be used to look at the reference and citation lists of all included full-text articles. This will highlight any additional articles not included in the database searches which may need to be considered for inclusion. Including citation lists is known as snowballing (or pearl growing) and considered an important aspect to ensure inclusivity of all relevant articles.[25] Gray literature will not be included as this is a niche topic with established international collaborations resulting in available research and literature reviews being published. The corresponding author for any article will be contacted if clarification is required for completeness of either including or excluding the article within the scoping review. The database searches will be peer-reviewed by the University Research Librarian for transparency and robustness of the search strategy.

SOURCE OF EVIDENCE SELECTION

Articles obtained will be stored on EndNote X9.2 and duplications removed. Initial screening of the studies will be done by reading the title and abstract to ascertain whether they fit the inclusion criteria. This screening will be carried out independently by two reviewers (HP and ASD) before the review team meet to discuss the articles to be included for full-text review. Any discrepancies will be resolved with the addition of the third reviewer (FG).

Full-text of the articles included based on abstract will be retrieved. These will be read in full by two reviewers (HP and ASD). Corresponding authors of the retrieved articles will be contacted for clarification if required. In the absence of being able to contact the corresponding author of an article, other members of the research team will be approached. The review team will meet again to discuss the final articles to be included in the scoping review and the third reviewer (FG) will resolve any discrepancies. Any full-text articles which are excluded at this stage will be justified in the scoping review report. For transparency the search results will be presented in the PRISMA flowchart (figure 1) which will systematically detail the selection process of articles.

For peer review only - http://bmjopen.bmj.com/site/about/guidelines.xhtml

DATA EXTRACTION

Data will be extracted from the articles obtained which meet the objectives of the scoping review. Data extraction will be undertaken in full by two reviewers (HP and ASD) and the third reviewer (FG) will randomly select 25% of the eligible articles to review. The third reviewer (FG) will review data extraction templates completed by the two full reviewers (HP and ASD) against the relevant article. This is a quality check to ensure the extracted data from the articles is accurate and is executed with rigour. Once the data extraction and review of the randomly selection articles has been completed independently by all three reviewers, the review team will meet to discuss and address any discrepancies.

For a consistent approach between reviewers, the Joanna Briggs Institute source of evidence template[23] will be modified to extract information, characteristics and findings from the articles which are relevant to answering the scoping review questions. This will include information on the population, concept, context, type of evidence source, study methods, values and preferences, information on what cancer-directed therapy was given and whether this was in conjunction with palliative care/symptom management. The modified data extraction template for this scoping review can be found in figure 5 and 6. Any amendments to the extraction template during the scoping review will be highlighted in the scoping review report.

ANALYSIS OF THE EVIDENCE

Analysis will commence with the final number of articles included in the scoping review and the basic characteristics of these articles. The data extraction template (figure 5 and 6) will highlight the most consistent and clear way for the evidence to be presented which is representative of the extracted data once the scoping review is complete. The data extraction template will record basic characteristics of the articles' such as first author, year of publication, country, study design (ie: qualitative, quantitative, mixed-methods, retrospective, prospective, longitudinal, cohort), research methods, and the number of participants in each study. This information will not be analysed but will be presented in the scoping review report to provide an overview of the included literature.

For qualitative data, content analysis will provide a description of data extracted and will be mapped to show consistencies and differences amongst the data.

For quantitative data, the frequency and numerical information will be presented based on the data extracted. For example, the sample size and types of study design could be individually displayed in graph or tabular format. In both the qualitative and quantitative data extraction, the analysis will reflect the objectives for the scoping review.

PRESENTATION OF RESULTS

Results will be presented in formats such as tabular, diagrammatic and descriptive summaries to clearly represent the extracted data relevant to the scoping review objectives. The basic characteristics of the studies will be presented in table format. This will include the citation, country, study design, methods and sample size of each article. This will provide the reader with an overall

For peer review only - http://bmjopen.bmj.com/site/about/guidelines.xhtml

summary of the studies which are included in the scoping review. Descriptive summaries of the qualitative data will be supported with visual diagrams mapping the relevant categories of the scoping review objectives. For example, a word diagram to illustrate the frequency of values and preferences which come from the data and subsequently mapping these concepts into themes to show inter-dependent relationships between these values and preferences. Quantitative data will be presented in graphs and figures to show comparisons and differences between the data extracted. The presentation of results may be refined during the scoping review to align with the data extracted from the included articles. Amendments to the suggested presentation will be highlighted in the scoping review report.

The scoping review will identify any research gaps which could enhance how support is provided to this parent population. Recommendations for practice which may support parents when making treatment decisions for cancer-directed therapy when their child has a poor-prognosis cancer will be highlighted.

ETHICS AND DISSEMINATION:

This scoping review is the first to look at parent values and preferences in treatment decision-making when their child has a poor-prognosis cancer. The results from this scoping review will be used to inform an interview schedule with parents to understand their values and preferences when making repeated treatment decisions for their child with relapse neuroblastoma, a poor prognosis childhood cancer. The combined results from this scoping review and parent interviews will inform the development of a decision aid to support parents in their treatment decision-making. This data and the decision aid developed will be published in peer-reviewed journals and presented at national/international conferences consisting of healthcare professionals, charities and parents. This scoping review does not require ethical approval.

For peer review only

For peer review only - http://bmjopen.bmj.com/site/about/guidelines.xhtml

REFERENCES

1. Steliarova-Foucher E, Colombet M, Ries LAG, et al. International incidence of childhood cancer, 2001-10: a population-based registry study. *Lancet Oncol* 2017;18(6):719-731. doi: 10.1016/S1470-2045(17)30186-9 [published Online first: 11 April 2017].
2. Childhood Cancer International. [Internet] 2020 [cited 2020 May 30]. Available from: https://www.childhoodcancerinternational.org/
3. Public Health England. Childhood Cancer Statistics, England Annual Report 2018. [Internet] 2018 [cited 2020 Apr 28]. Available from: http://www.ncin.org.uk/publications/reports
4. Cancer Research UK. Children's cancer statistics. [Internet] 2019 [cited 2020 Apr 28]. Available from:https://www.cancerresearchuk.org/health-professional/cancer-statistics/childrens-cancers
5. Mack JW, Feudtner C, Hinds PS. Communication and decision support for children with advanced cancer and their families. *Am Soc Clin Oncol Educ Book*. 2012;32:637-43. doi: 10.14694/EdBook_AM.2012.32.164. [published Online first: 17 May, 2012].
6. Hinds PS, Oakes L, Furman W, et al. Decision making by parents and healthcare professionals when considering continued care for pediatric patients with cancer. *Oncol Nurs Forum* 1997;24(9):1523-28.
7. Lynch A, Murphy A. The emerging role of immunotherapy in colorectal cancer. *Ann Transl Med* 2016;4(16). doi: 10.21037/atm.2016.08.29.
8. Somasundaram A, Burns TF. The next generation of immunotherapy: keeping lung cancer in check. *J Hematol Oncol* 2017;10(1):87. doi: 10.1186/s13045-017-0456-5.
9. Mackall CL, Merchant MS, Fry TJ. Immune-based therapies for childhood cancer. *Nat Rev Clin Oncol* 2014;11:693–703. doi: 10.1186/s13045-017-0456-5. [published Online first: 28 October 2014].
10. Roberts SS, Chou AJ, Cheung N-KV. Immunotherapy of childhood sarcomas. *Front Oncol* 2015;5:181. doi: 10.3389/fonc.2015.00181.
11. National Cancer Institute. Understanding Cancer Prognosis. [Internet] 2019 [cited 2020 Mar 31]. Avaliable from: https://www.cancer.gov/about-cancer/diagnosis-staging/prognosis
12. Lichtenthal WG, Roberts KE, Catarozoli C, et al. Regret and unfinished business in parents bereaved by cancer: A mixed methods study. *J Palliat Med* 2020;34(3):367-377. doi: 10.1177/0269216319900301. [published Online first: 5 February 2020].
13. Lannen PK, Wolfe K, Prigerson HG, et al. Unresolved grief in a national sample of bereaved parents: impaired mental and physical health 4 to 9 years later. *J Clin Oncol* 2008;26(36):5870-5876. doi: 10.1200/JCO.2007.14.6738. [published Online first: 24 November 2008].
14. Tomlinson D, Bartels U, Gammon J, et al. Chemotherapy versus supportive care alone in pediatric palliative care for cancer: comparing the preferences of parents and healthcare professionals. *Can Med Assoc J* 2011;183(7):E1252-58. doi: 10.1503/cmaj.110392. [published Online first: 17 October 2011].

For peer review only - http://bmjopen.bmj.com/site/about/guidelines.xhtml

15. Robertson EG, Wakefield CE, Shaw J, et al. Decision-making in childhood cancer: parents' and adolescents' views and perceptions. *Support Care Cancer* 2019;27:4331-4340. doi: 10.1007/s00520-019-04728-x.
16. Kaye E, Mack JW. Parent perceptions of the quality of information received about a child's cancer. *Pediatr Blood Cancer* 2013;60:1896-1901. doi: 10.1002/pbc.24652.
17. Kilicarslan-Toruner E, Akgun-Citak E. Information-seeking behaviours and decision-making process of parents of children with cancer. *Eur J Oncol Nurs* 2013;17(2):176-183. doi: 10.1016/j.ejon.2012.03.001.
18. Straus SE, Richardson WS, Glasziou P, et al. Evidence-Based Medicine How to Practice and Teach EBM. 3rd ed. Edinburgh: Elsevier Churchill Livingstone 2005:12.
19. Marron JM, Mack JW. When to say when: How aggressively to care for children with multiply relapsed cancer?. *Pediatr Blood Cancer* 2015;62(7):1119-20. doi: 10.1002/pbc.25476. [published Online first: 8 March 2015].
20. Markward MJ, Benner K, Freese R. Perspectives of parents on making decisions about the care and treatment of a child with cancer: A review of the literature. *Fam Syst Health* 2013;31(4):406-413. doi: 10.1037/a0034440.
21. Valdez-Martinez E, Noyes J, Bedolla M. When to stop? Decision-making when children's cancer treatment is no longer curative: a mixed methods systematic review. *BMC Pediatr* 2014;4(24):124. doi: 10.1186/1471-2431-14-124.
22. Hinds PS, Birenbaum LK, Clarke-Steffen L. Coming to terms: Parents' response to a first cancer recurrence in their child. *Nurs Res* 1996;45(3):148-153. Doi: 10.1097/00006199-199605000-00005.
23. Peters MDJ, Godfrey C, McInerney P, et al. Chapter 11: Scoping Reviews (2020 version). In: Aromataris E, Munn Z. (Editors) Joanna Briggs Institute Reviewer's Manual, JBI, 2020. Available from: https://reviewersmanual.joannabriggs.org/
24. Tricco, AC, Lillie E, Zarin W, et al. PRISMA Extension for Scoping Reviews (PRISMA-ScR): Checklist and Explanation. *Ann Intern Med* 2018;169(7): 467-73. doi: 10.7326/M18-0850. [published Online first: 4 September 2018].
25. Greenhalgn T, Peacock R. Effectiveness and efficiency of search methods in systematic reviews of complex evidence: audit of primary sources. *BMJ* 2005; 331:1064-1065. doi: 10.1136/bmj.38636.593461.68. [published Online first: 17 October 2005].

PATIENT AND PUBLIC INVOLVEMENT

No patient involvement for this scoping review.

AUTHOR CONTRIBUTIONS

HP, FG, ASD conceived the idea for this scoping review. HP developed the research questions, objectives, inclusion criteria, undertook initial database search, drafted and edited this manuscript. FG and ASD reviewed inclusion criteria, screened abstracts and full-text papers from initial database search, and edited this manuscript. HP, FG, ASD approved final manuscript version.

For peer review only - http://bmjopen.bmj.com/site/about/guidelines.xhtml

FUNDING STATEMENT:

Helen Pearson is supported by National Institute Health Research (NIHR) Clinical Doctoral Research Fellowship NIHR300548.

ACKNOWLEDGEMENTS:

This research was supported in part by the NIHR Great Ormond Street Hospital Biomedical Research Centre.

COMPETING INTEREST STATEMENT

None declared

FIGURE LEGENDS

Figure 1: PRISMA Flowchart

Figure 2, 3 and 4: Medline Search Strategy

Figure 5 and 6: JBI Data Extraction Tool

For peer review only

For peer review only - http://bmjopen.bmj.com/site/about/guidelines.xhtml

Figure 1. Preferred Reporting Items for Systematic Reviews and Meta-Analyses (PRISMA) flowchart for the scoping review

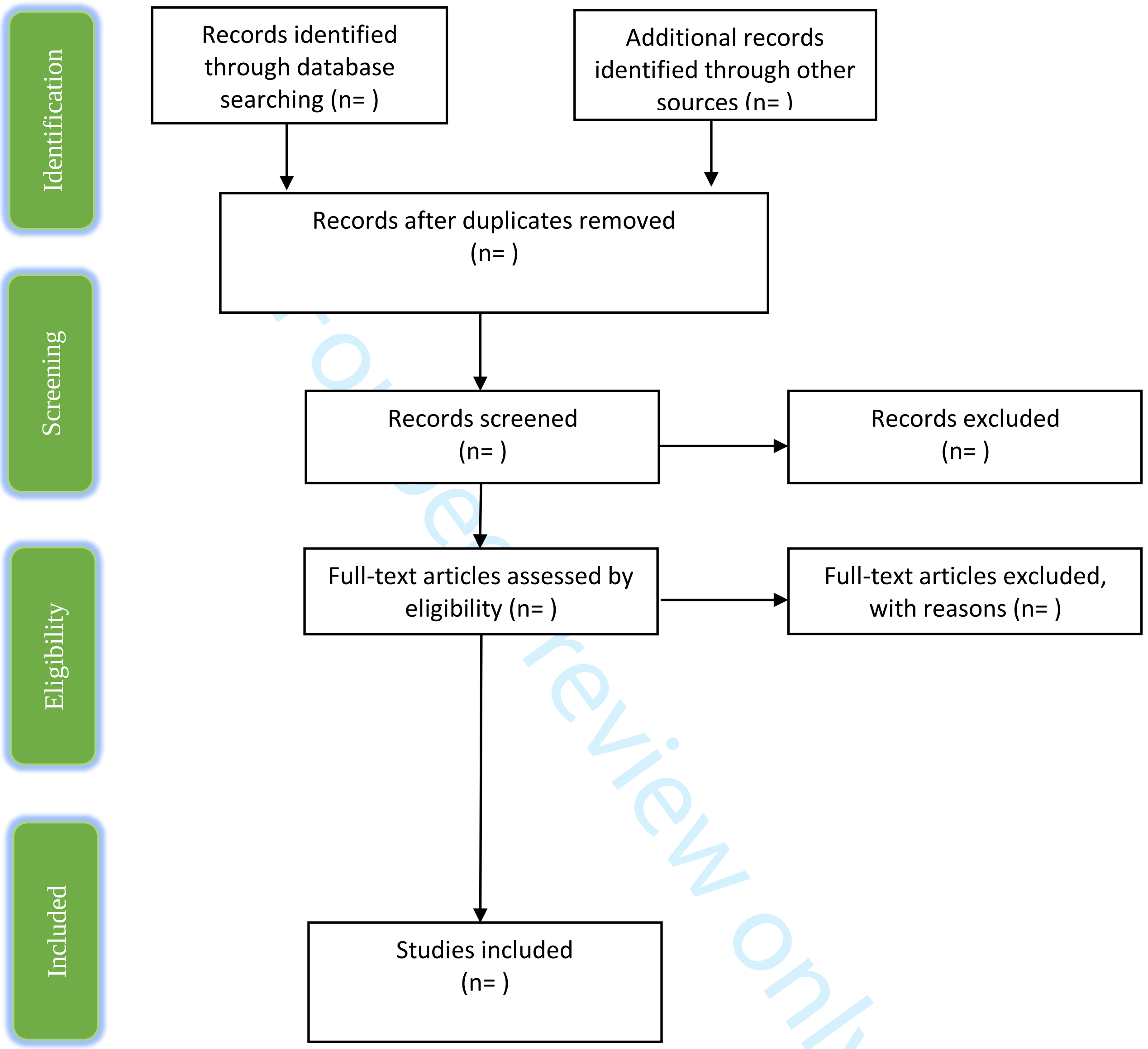

Figure 2: Medline Search Strategy

Medline Search Strategy
S1 parent*
S2 mother*
S3 father*
S4 famil*
S5 S1 OR S2 OR S3 OR S4
S6 value*
S7 (MH "Value of Life") OR (MH "Social Values")
S8 preference*
S9 "goal* of care"
S10 choice*
S11 belief*
S12 (MH "Social Norms")
S13 attitude*
S14 expect*
S15 (MH "Motivation")
S16 predisposition*
S17 influenc*
S18 experienc*
S19 (MH "Clinical Decision-Making")
S20 choose*
S21 percept*
S22 S6 OR S7 OR S8 OR S9 OR S10 OR S11 OR S12 OR S13 OR S14 OR S15 OR S16 OR S17 OR S18 OR S19 OR S20 OR S21

For peer review only

Figure 3: Medline Search Strategy

S23 "decision making"
S24 (MH "Decision Making, Shared")
S25 decision N2 making
S26 decision*
S27 decide*
S28 S23 OR S24 OR S25 OR S26 OR S27
S29 S5 AND S22 AND S28
S30 "poor prognosis"
S31 relaps*
S32 (MH "Recurrence")
S33 recurrenc*
S34 refractory
S35 incurable
S36 (MH "Terminally Ill")
S37 "advanc* cancer"
S38 "no realistic chance of cure"
S39 uncertain*
S40 deteriorat*
S41 (MH "Clinical Deterioration")
S42 "disease* progress*"
S43 "palliat* chemotherap*"
S44 "no reasonable chance of cure"
S45 S30 OR S31 OR S32 OR S33 OR S34 OR S35 OR S36 OR S37 OR S38 OR S39 OR S40 OR S41 OR S42 OR S43 OR S44
S46 child*

For peer review only

Figure 4: Medline Search Strategy

S47	p#ediatric*
S48	S46 OR S47
S49	cancer
S50	neoplasm*
S51	(MH "Neoplasm Recurrence, Local")
S52	malignan*
S53	(MH "Neoplasms")
S54	"cancer direct* therap*"
S55	oncolog*
S56	S49 OR S50 OR S51 OR S52 OR S53 OR S54 OR S55
S57	S45 AND S48 AND S56
S58	S29 AND S57

For peer review only

Figure 5: JBI Data Extraction Template amended for this Scoping Review

Scoping Review Details	
Scoping Review Title	
Review Objectives	
Review Questions	
Inclusion/Exclusion Criteria	
Population (parent of a child under age of 18 years)	
Concept (parent of a child receiving cancer-directed therapy for a poor prognosis cancer)	
Context (hospital, community, hospice)	
Types of Evidence Source (Qual/Quant, mixed methods)	
Evidence source Details and Characteristics	
Citation details (author, year of publication, journal)	
Country	
Study Aims/Objectives	

For peer review only

Figure 6: JBI Data Extraction Template amended for this Scoping Review

Prospective or Retrospective study	
Longitudinal or Cohort study	
Research Methodology & Methods	
Participant Details (mother,father sample size)	
Data extracted from sources of evidence	
Cancer-directed therapy options available (list)	
Cancer-directed therapy in conjunction with palliative care/symptoms management	YES / NO
Cancer-directed therapy given to child (list if more than one)	
Outcome from cancer-directed therapy given	
Values and preferences identified (list)	
Explanation of how these values and preferences informed decision-making (explicitly stated/not stated/unsure)	
Article recommendations for future research/clinical practice	

For peer review only

Supplementary Figure 1: Preferred Reporting Items for Systematic reviews and Meta-Analyses extension for Scoping Reviews (PRISMA-ScR) Checklist

SECTION	ITEM	PRISMA-ScR CHECKLIST ITEM	REPORTED ON PAGE #
**TITLE**			
Title	1	Identify the report as a scoping review.	Page 1 Line 1
**ABSTRACT**			
Structured summary	2	Provide a structured summary that includes (as applicable): background, objectives, eligibility criteria, sources of evidence, charting methods, results, and conclusions that relate to the review questions and objectives.	Page 2 Lines 1-26
**INTRODUCTION**			
Rationale	3	Describe the rationale for the review in the context of what is already known. Explain why the review questions/objectives lend themselves to a scoping review approach.	Page 3-5 Lines 41-140
Objectives	4	Provide an explicit statement of the questions and objectives being addressed with reference to their key elements (e.g., population or participants, concepts, and context) or other relevant key elements used to conceptualize the review questions and/or objectives.	Page 5-6 Lines 141-181
**METHODS**			
Protocol and registration	5	Indicate whether a review protocol exists; state if and where it can be accessed (e.g., a Web address); and if available, provide registration information, including the registration number.	Page 6 Lines 184-185
Eligibility criteria	6	Specify characteristics of the sources of evidence used as eligibility criteria (e.g., years considered, language, and publication status), and provide a rationale.	Page 6 Lines 166-181
Information sources*	7	Describe all information sources in the search (e.g., databases with dates of coverage and contact with authors to identify additional sources), as well as the date the most recent search was executed.	Page 6-7 Lines 191-205
Search	8	Present the full electronic search strategy for at least 1 database, including any limits used, such that it could be repeated.	Page 7-8 Lines 208-221 Appendix Figure 2
Selection of sources of evidence†	9	State the process for selecting sources of evidence (i.e., screening and eligibility) included in the scoping review.	Page 8 Lines 239-253
Data charting process‡	10	Describe the methods of charting data from the included sources of evidence (e.g., calibrated forms or forms that have been tested by the team before their use, and whether data charting was done independently or in duplicate) and any processes for obtaining and confirming data from investigators.	Page 9 Lines 255-271
Data items	11	List and define all variables for which data were sought and any assumptions and simplifications made.	N/A

For peer review only

SECTION	ITEM	PRISMA-ScR CHECKLIST ITEM	REPORTED ON PAGE #
Critical appraisal of individual sources of evidence§	12	If done, provide a rationale for conducting a critical appraisal of included sources of evidence; describe the methods used and how this information was used in any data synthesis (if appropriate).	N/A
Synthesis of results	13	Describe the methods of handling and summarizing the data that were charted.	Page 9-10 Lines 271-307
**RESULTS**			
Selection of sources of evidence	14	Give numbers of sources of evidence screened, assessed for eligibility, and included in the review, with reasons for exclusions at each stage, ideally using a flow diagram.	N/A
Characteristics of sources of evidence	15	For each source of evidence, present characteristics for which data were charted and provide the citations.	N/A
Critical appraisal within sources of evidence	16	If done, present data on critical appraisal of included sources of evidence (see item 12).	N/A
Results of individual sources of evidence	17	For each included source of evidence, present the relevant data that were charted that relate to the review questions and objectives.	N/A
Synthesis of results	18	Summarize and/or present the charting results as they relate to the review questions and objectives.	N/A
**DISCUSSION**			
Summary of evidence	19	Summarize the main results (including an overview of concepts, themes, and types of evidence available), link to the review questions and objectives, and consider the relevance to key groups.	NA/
Limitations	20	Discuss the limitations of the scoping review process.	N/A
Conclusions	21	Provide a general interpretation of the results with respect to the review questions and objectives, as well as potential implications and/or next steps.	N/A
**FUNDING**			
Funding	22	Describe sources of funding for the included sources of evidence, as well as sources of funding for the scoping review. Describe the role of the funders of the scoping review.	Page 13

JBI = Joanna Briggs Institute; PRISMA-ScR = Preferred Reporting Items for Systematic reviews and Meta-Analyses extension for Scoping Reviews.

* Where *sources of evidence* (see second footnote) are compiled from, such as bibliographic databases, social media platforms, and Web sites.

† A more inclusive/heterogeneous term used to account for the different types of evidence or data sources (e.g., quantitative and/or qualitative research, expert opinion, and policy documents) that may be eligible in a scoping review as opposed to only studies. This is not to be confused with *information sources* (see first footnote).

‡ The frameworks by Arksey and O'Malley (6) and Levac and colleagues (7) and the JBI guidance (4, 5) refer to the process of data extraction in a scoping review as data charting.

§ The process of systematically examining research evidence to assess its validity, results, and relevance before using it to inform a decision. This term is used for items 12 and 19 instead of "risk of bias" (which is more applicable to systematic reviews of interventions) to include and acknowledge the various sources of evidence that may be used in a scoping review (e.g., quantitative and/or qualitative research, expert opinion, and policy document).

For peer review only
